# Plant Disease and Phytopathogen Research in Central Asia: A Bibliometric Mapping of Research Trends, Collaboration, and Emerging Directions

**DOI:** 10.3390/microorganisms14102358

**Published:** 2026-10-15

**Authors:** Aziza Usmonkulova, Ilkhom Khalilov, Mukhiddin Juliev, Nigora Tillyaxodjayeva, Rixsiniso Karimova, Asror Rakhmatov, Dilfuza Tirkashboyevna Turdiyeva, Nafosat Kurbonova, Tolib Rajabov, Zukhra Yu. Akhmedova, Nigora Y. Zukhritdinova, Feruzjon Shodmonov, Oysha Jabborova, Amir Alimbetov

**Affiliations:** 1 Scientific Research Institute of the Plant Quarantine and Protection, St. Bobur 4, Tashkent 111215, Uzbekistan; 2 Institute of Microbiology of Academy Sciences of the Republic of Uzbekistan, St. A. Kadyri 7B, Tashkent 100128, Uzbekistan; ilkhom2002@yahoo.com; 3 Institute of Fundamental and Applied Research, “TIIAME” National Research University, Kary-Niyaziy Str. 39, Tashkent 100000, Uzbekistan; mukhiddinjuliev@gmail.com; 4 Department of Civil Engineering and Architecture, Turin Polytechnic University in Tashkent, St. Little Ring Road 17, Tashkent 100095, Uzbekistan; 5 Department of “Life Safety”, Tashkent State Technical University, St. Universitet 2, Tashkent 100095, Uzbekistan; 6 Department of Agricultural Phytopathology, Tashkent State Agrarian University, St. University 4, Tashkent 111218, Uzbekistan; 7 Department of Preventive Medicine, Termez University of Economics and Service, St. Ibn Sino 38-B, Termez 190111, Uzbekistan; 8 Institute of Zoology, Academy of Sciences of the Republic of Uzbekistan, St. Bogishamol 232 B, Tashkent 100053, Uzbekistan; 9 Department of Biotechnology and Food Safety, Bukhara State University, St. Muhammad Iqbal Street, 11, Bukhara 705018, Uzbekistan; 10 Bukhara State Medical Institute Named After Abu Ali Ibn Sina, St. Gijduvon, 23, Bukhara 200118, Uzbekistan; 11 Department of Ecology and Soil Science, Karakalpak State University, St. Ch. Abdirov 1, Nukus 230112, Uzbekistan

**Keywords:** bibliometric analysis, plant diseases, phytopathogenic threats, Central Asia, Scopus, VOSviewer, Bibliometrix, sustainable disease management

## Abstract

Plant diseases and phytopathogenic threats pose significant challenges to agricultural productivity and food security in Kazakhstan, Kyrgyzstan, Tajikistan, Turkmenistan, and Uzbekistan. This study aimed to characterize research development, scientific productivity, collaboration patterns, intellectual structure, and emerging trends in plant disease and phytopathogen research. A bibliometric analysis was conducted using 505 Scopus-indexed publications from 1994 to 2026, covering 253 sources and 2485 authors. Bibliometrix/Biblioshiny and VOSviewer were used for performance analysis and science mapping, including citation, co-authorship, collaboration, co-citation, bibliographic coupling, historiograph, and keyword co-occurrence analyses. Research output showed an annual growth rate of 11.65%, with an average of 13.94 citations per publication. Kazakhstan (143 publications) and Uzbekistan (82 publications) were the leading contributors, while international co-authorship accounted for 33.66% of publications. Three major research directions were identified: wheat disease resistance and molecular breeding, plant pathogens and fungal biodiversity, and plant-associated microorganisms, microbiology, and phylogenetic characterization. Recent trends indicate increasing attention to molecular and genomic approaches, microbiome research, and deep learning. Overall, research in Central Asia is becoming increasingly multidisciplinary, highlighting the need to integrate genomic diagnostics, pathogen surveillance, molecular breeding, biological control, and data-driven approaches for sustainable plant disease management.

## 1. Introduction

Agricultural production in Central Asia is increasingly affected by plant diseases that threaten crop productivity, food security, and the sustainability of agricultural systems. The region relies heavily on wheat and other staple crops, making plant disease research particularly important for regional agricultural resilience. Previous studies have documented diverse phytopathological challenges across Kazakhstan, Kyrgyzstan, Tajikistan, Turkmenistan, and Uzbekistan, indicating a broad and multidisciplinary research field encompassing pathogen diversity, disease epidemiology, host resistance, and disease management [1,2,3,4,5,6,7].

The Central Asian region also faces challenges associated with disease surveillance, pathogen detection, and phytosanitary capacity. Effective surveillance and early detection are essential for identifying emerging pathogens before they become widely established. Global disease surveillance frameworks have emphasized the importance of coordinated monitoring, standardized diagnostic approaches, and timely information exchange for reducing the risks associated with emerging crop diseases [8,9,10]. Recent studies from Central Asia further demonstrate the value of molecular approaches for pathogen identification and characterization. For example, genomic and molecular analyses have been used to investigate the introduction patterns of *Erwinia amylovora*, while molecular markers have been applied to identify resistance genes in wheat and characterize pathogen populations [3,7]. These developments indicate a gradual transition from conventional disease diagnosis toward more precise molecular and genetic approaches.

Beyond direct yield losses, plant diseases can affect food security, agricultural livelihoods, and ecosystem stability [11,12,13]. These broader impacts further emphasize the importance of understanding how plant disease research has developed across the region.

Although research on plant diseases and phytopathogenic threats in Central Asia has expanded considerably, it remains distributed across several interconnected disciplines, including plant pathology, crop breeding, fungal taxonomy, microbiology, molecular biology, and agricultural biotechnology. Existing studies have generally focused on individual crops, pathogens, diseases, or countries, whereas the broader structure and evolution of the regional research landscape remain insufficiently characterized. In particular, limited evidence is available regarding the temporal development of research productivity, the contributions of countries, institutions, and authors, the structure of research collaboration, and the emergence or decline of thematic areas. A systematic bibliometric assessment can address these gaps by providing an integrated view of the development and structure of the field.

Bibliometric analysis provides a quantitative framework for addressing these gaps by combining publication and citation indicators with science-mapping techniques. It can reveal publication and citation development, research collaboration structures, intellectual relationships among publications and research communities, and the evolution of major research themes [14,15]. Applying these approaches to Central Asian plant disease research can therefore provide a broader perspective than disease-specific reviews and identify major and emerging directions in the field.

Therefore, this study aimed to characterize the development and structure of plant disease and phytopathogen research in Central Asia using a bibliometric approach. Specifically, we examined (i) temporal publication and citation trends; (ii) contributions of journals, authors, countries, and institutions; (iii) regional and international collaboration patterns; (iv) the intellectual structure of the field; and (v) dominant, emerging, and evolving research themes using bibliometric and thematic analyses.

To our knowledge, no previous bibliometric study has comprehensively mapped the development, intellectual structure, collaboration patterns, and thematic evolution of plant disease and phytopathogen research across the five Central Asian countries using a longitudinal Scopus dataset. The present study addresses this gap by integrating performance analysis, co-authorship, co-citation, bibliographic coupling, historiographic analysis, and thematic evolution over the period 1994–2026.

## 2. Materials and Methods

### 2.1. Database Selection and Search Strategy

There are various bibliometric analysis tools like Bibexcel, BiblioMaps, SciMAT, Cit NetExplorer, VOSviewer, Sci2 Tool, CiteSpace, and Biblioshiny available for analyzing bibliometric data, each having its own strengths and capabilities. In this study, Bibliometrix (version 4.6.0.), an R package used for conducting bibliometric analysis using R-Studio software with an inbuilt Biblioshiny (2026, Naples, Italy, version 4.6.0), was used. Biblioshiny is a web-based interface system that adds further functionalities that enhance user-friendliness, offer several tools for analysis, and present numerous unique visualizations [14]. Its main limitation is that the quality and interpretability of the resulting indicators depend on the completeness, consistency, and standardization of the underlying bibliographic metadata. VOSViewer (2023, Leiden, The Netherlands, version 1.6.20) is another tool used in this study for generating and exploring maps to visualize bibliometric network data on several types of analyses like co-authorship, co-occurrence, citation, bibliographic coupling, and co-citation linkages [15]. Its principal limitation is that network structure and clustering may depend on the selected thresholds, counting procedures, normalization settings, and quality of the input metadata. The Biblioshiny and VOSViewer tools were fed with the CSV file from the Scopus database to produce visual plots for several analyses conducted in this study. They also enable comparisons between the results for similar types of analyses from both applications.

There are different academic databases for the analysis of bibliometric data like Web of Science (WoS), Google Scholar, Di mensions, and Microsoft Academic. Nonetheless, for this particular paper, Scopus was selected for the purpose of data retrieval due to its higher coverage of articles published in peer-reviewed journals and conference proceedings, as well as higher-quality metadata [16]. Despite the fact that choosing a particular database might lead to biases, previous comparative studies performed by analyzing bibliometric databases show that Scopus usually indexes greater coverage with better and more convenient access to data [17,18]. Scopus provides extensive coverage of peer-reviewed journals and other scholarly sources and enables systematic analysis of publication productivity, citation performance, collaboration patterns, and thematic structures.

In this regard, according to a study done by Singh et al., Scopus indexes about 66.07% more unique journals than WoS [19]. Therefore, Scopus is widely used in bibliometric analysis papers, as can be seen from different studies, for instance, Kabir et al., Slimani et al., Yang et al., and Usmonkulova et al. [20,21,22,23]. The four steps of conducting a bibliometric analysis proposed by Donthu et al. have been considered in this study [24]. Although the broader overall coverage of Scopus was an important consideration in its selection as the primary bibliographic database, it was not assumed to provide comprehensive coverage of plant pathology research conducted across Central Asia.

The present study was therefore designed to characterize the Scopus-indexed research landscape rather than to estimate the complete volume of plant pathology research produced in the region. Accordingly, the findings should be interpreted within the scope of the database and search strategy used in this study.

It is necessary to point out that the relevance of choosing bibliometric analysis criteria cannot be underestimated. They include selecting databases to use for this purpose, the timeframe within which research was conducted, and choosing keywords to consider. Thus, a list of keywords was suggested as the query string, and “AND,” “OR” logical operators were used in order to define the research field. Moreover, an asterisk (*) was used as a substitute for any variations at the end of the keyword. Following preliminary testing of alternative keyword combinations, the final search strategy was established and used for the retrieval of the dataset analyzed in this study. The search was conducted in Scopus using the TITLE-ABS-KEY field, which searches article titles, abstracts, and author keywords. The complete final search string is provided below to ensure reproducibility:

TITLE-ABS-KEY ( ( Kazakhstan OR Uzbekistan OR Kyrgyzstan OR Tajikistan OR Turkmenistan OR Kazakh* OR Uzbek* OR Kyrgyz* OR Tajik* OR Turkmen* ) AND ( “plant disease*” OR “crop disease*” OR phytopatholog* OR phytopathogen* OR “plant pathogen*” OR “crop pathogen*” OR “plant pest*” OR “plant health” OR “plant protection” OR phytosanitary OR quarantine OR “plant biosecurity” OR “fungal pathogen*” OR Fusarium OR Alternaria OR Puccinia OR Ustilago OR Rhizoctonia OR Verticillium OR Phytophthora OR Pythium OR Botrytis OR Colletotrichum OR Xanthomonas OR “Pseudomonas syringae” OR Erwinia OR Ralstonia OR “plant virus*” OR phytoplasma* OR “plant parasitic nematode*” ) ) AND NOT TITLE-ABS-KEY ( COVID-19 OR SARS-CoV-2 OR coronavirus OR patient* OR hospital* OR clinical* OR medicine OR cancer OR diabetes OR HIV OR tuberculosis ) AND ( LIMIT-TO ( LANGUAGE, “English” ) ).

The search strategy was not limited to the generic terms “plant disease” or “phytopathogen”. It also incorporated multiple pathogen-specific terms, including *Fusarium*, *Alternaria*, *Puccinia*, *Ustilago*, *Rhizoctonia*, *Verticillium*, *Phytophthora*, *Pythium*, *Botrytis*, *Colletotrichum*, *Xanthomonas*, *Pseudomonas syringae*, *Erwinia*, *Ralstonia*, phytoplasma, and plant parasitic nematode. Viral research was represented through the “plant virus*” term. 

The search was conducted on 6 July 2026 and covered publications from 1994 through 6 July 2026. The search was restricted to English-language records using the Scopus language filter. No additional publication-year restriction was applied. The query incorporated alternative country-name forms using wildcard operators and included both broad plant-health concepts and a range of specific fungal, bacterial, viral, phytoplasma, and nematode-related terms. 

### 2.2. Eligibility and Screening Criteria

Records were considered eligible when they met the following criteria: (i) they were indexed in Scopus; (ii) they were published in English; (iii) they were published between 1994 and 6 July 2026; and (iv) their title, abstract, or author keywords contained terms indicating a geographical connection with Kazakhstan, Uzbekistan, Kyrgyzstan, Tajikistan, or Turkmenistan and a relevant plant disease, phytopathogen, plant pathogen, plant health, plant protection, phytosanitary, quarantine, or related pathogen-specific topic included in the predefined search strategy. Records retrieved through the search but not meeting these criteria were excluded. Duplicate or inconsistent bibliographic records identified during data cleaning were also excluded from the final analytical dataset. No additional country, institution, pathogen, or publication-specific manual selection was applied after the predefined search strategy.

### 2.3. Data Extraction and Preprocessing

**Figure 1:**
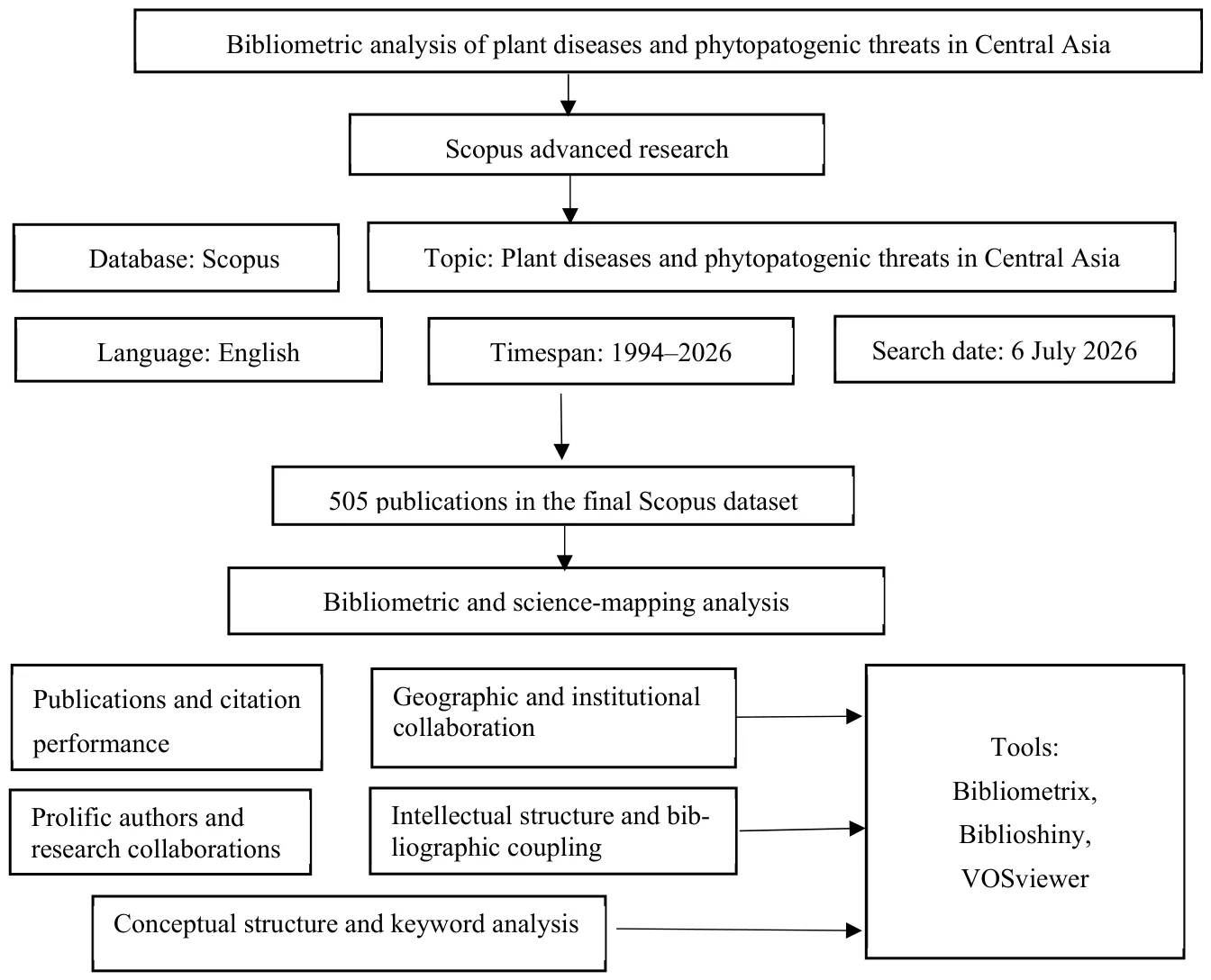
Research design and workflow of the bibliometric analysis of plant diseases and phytopathogenic threats in Central Asia.

After applying the exclusion criteria, a search of the Scopus database yielded 505 results. The Scopus records were exported in CSV format and checked for duplicates and inconsistencies in bibliographic metadata. Author names, institutional affiliations, country names, and keywords were standardized where necessary. Duplicate records were assessed using DOI, title, author, and publication-year information. The cleaned dataset was subsequently used for all bibliometric analyses (Figure 1).

### 2.4. Bibliometric Indicators

Bibliometric performance was assessed using annual publication output, citation counts, average citations per document, source productivity, h-index, author productivity, country contributions, single-country publications (SCP), multiple-country publications (MCP), and international co-authorship. These indicators were used to evaluate publication performance and research impact.

### 2.5. Network Analysis and Visualization

Science mapping was performed using VOSviewer version 1.6.20 and Bibliometrix version 4.6.0. The analyses included co-authorship, citation, co-citation, bibliographic coupling, and keyword co-occurrence analyses. Network maps were generated to examine collaboration patterns, intellectual structures, and major research themes within the Scopus-indexed literature. Author and country collaboration were assessed using co-authorship networks, while bibliographic coupling and co-citation analyses were used to identify relationships among publications, sources, and knowledge structures. Co-word analysis was performed using author keywords.

The unit of analysis was selected according to the objective of each analysis and included authors, countries, organizations, publication sources, documents, references, and author keywords. Network-specific thresholds were applied where specified. For organization-level collaboration analysis, organizations were required to have a minimum of 5 documents and 4 citations, resulting in 13 organizations and six collaboration clusters. For source co-citation analysis, a minimum threshold of 20 citations per source was applied. For bibliographic coupling of publications, a minimum threshold of 30 citations per document was used. The author co-authorship analysis included 49 authors distributed across nine collaboration clusters, while the keyword analysis included 50 selected keywords for visualization.

## 3. Results

This section presents and discusses the results of the bibliometric analysis in relation to the research questions addressed in the study. The findings are interpreted through the performance analysis of various research constituents, employing publication, citation, and combined publication-citation indicators as proposed by Passas [25]. A total of 505 publications were analyzed, including 390 journal articles, 24 reviews, 19 book chapters, and 72 conference papers published between 1994 and 2026, demonstrating a substantial annual growth rate of 11.65%. The growing significance of plant diseases and phytopathogenic threats in Central Asia is reflected by an average of 13.94 citations per publication and a cumulative total of 87,610 references. Furthermore, 2485 authors contributed to research on plant disease management, with an average of 6.3 co-authors per publication. International co-authorship accounted for 33.66% of the publications, indicating that approximately one-third of the publications involved international collaboration. The descriptive statistics of the study are presented in Table 1.

### 3.1. Overall Publication and Citation Performance

**Figure 2:**
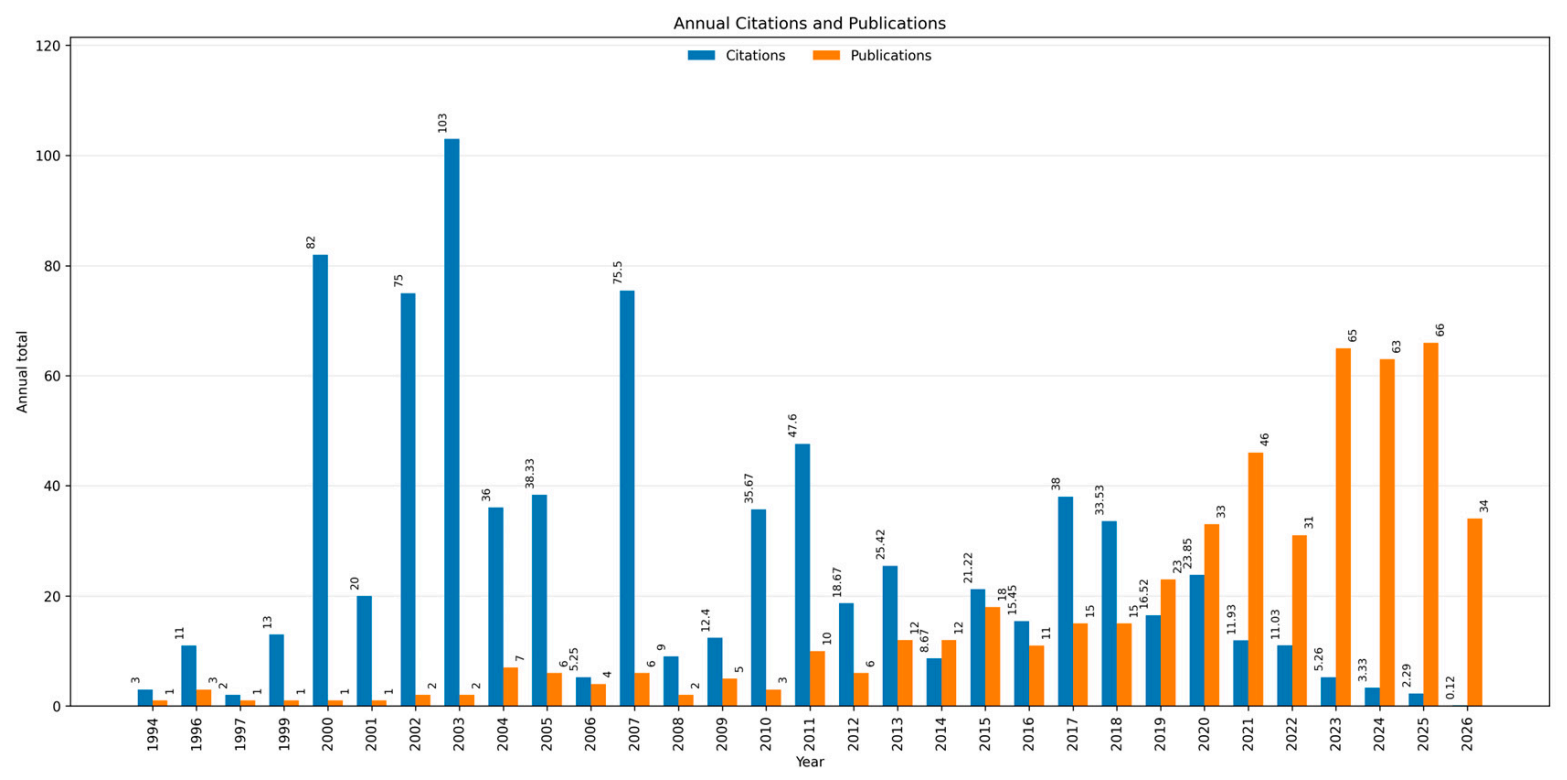
Annual publication and citation trends in plant disease research.

The annual papers addressing Central Asian countries trend illustrated in Figure 2 highlights the rapid development of research in plant diseases and phytopathogenic threats. The temporal distribution of publications demonstrates a clear long-term growth in research activity over the study period (1994–2026). The publication output remained relatively limited during the early years, with fewer than 10 publications annually before 2011. A gradual increase was observed from 2011 onward, followed by a marked acceleration after 2019. The number of publications increased from 23 in 2019 to 33 in 2020 and reached 46 in 2021, before rising sharply to 65 in 2023 and 66 in 2025, representing the highest annual output during the analyzed period.

In contrast, annual citation counts were considerably higher in the earlier years, reaching a maximum of 103 citations in 2003. Subsequently, citation rates generally declined despite the substantial increase in publication output. This pattern is typical of recent bibliometric records, as newly published studies have had less time to accumulate citations. Overall, the simultaneous increase in publications and decline in annual citations indicates a substantial expansion of research activity in recent years, with the field becoming increasingly active and productive.

Total citations and impact indicators such as the h-index are widely used in bibliometric studies to determine the most influential publication sources. These metrics offer valuable information regarding how frequently publications are cited, thereby reflecting their significance and academic influence within a specific research domain. Table 2 summarizes the leading journals contributing to plant disease research in Central Asia based on the number of published articles, h-index based on Scopus data, and total citations (TC). *E3S Web of Conferences* has the highest number of published articles in this field (23), followed by *Online Journal of Biological Sciences* (17), *Acta Horticulturae* (14), and *Bio Web of Conferences* (14). However, journals with fewer publications showed much higher citation impact. In particular, *Plant Disease* published 12 articles but received the highest number of total citations (TC—238), indicating a relatively strong scientific impact in the dataset. Similarly, *Acta Horticulturae* received 177 citations from 14 articles, while *Online Journal of Biological Sciences* received 107 citations from 17 articles.

The h-index further highlights the differences in research impact between journals. *Plants* showed the highest h-index (146), followed by *Plant Disease* (141), *Acta horticulturae* (77), *Journal of Plant Pathology* (62) and E3S Web of Conferences (52).

Overall, the results show that publications consistently differ in their effectiveness and impact on citations. Conference proceedings, such as *E3S Web of Conferences* and *Bio Web of Conferences*, accounted for a relatively large proportion of publications but showed relatively low citation accumulation (TC—22 and 15, respectively). In contrast, plant pathology and agricultural journals, in particular *Plant Disease*, *Acta Horticulturae*, *Plants*, and *Journal of Plant Pathology*, showed relatively high citation rates. This situation suggests that research on Central Asian plant diseases has gained more visibility when published in specialized and internationally recognized journals, while a large part of the regional literature has been concentrated in conference proceedings.

**Figure 3:**
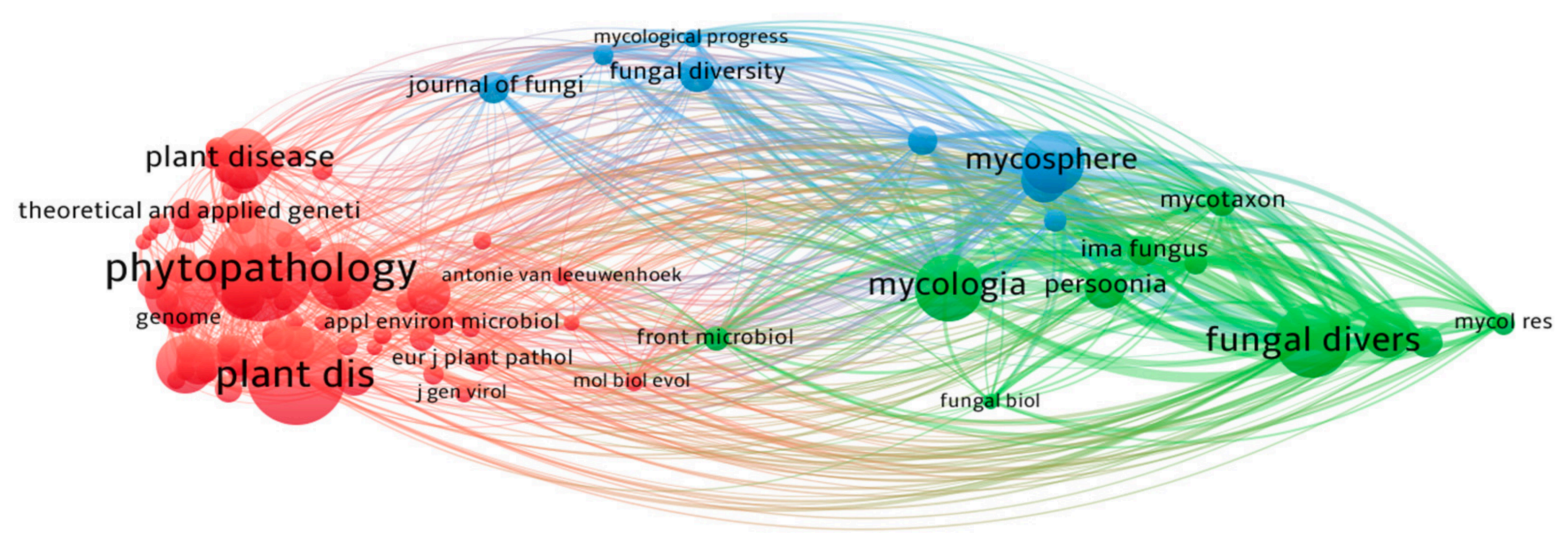
Network visualization of the most impactful publication sources based on co-citation network generated by VOSViewer (2023, Leiden, The Netherlands, version 1.6.20). Different colors represent distinct keyword clusters in the co-occurrence network.

The evaluation of influential publication sources can further be supported through science mapping techniques, which illustrate the structural relationships among journals. Figure 3 presents a VOSviewer-generated visualization of publication source influence in research on plant diseases and phytopathogenic threats based on co-citation networks. A minimum citation threshold of 20 citations per source was applied. These sources were organized into three distinct clusters, reflecting the major knowledge domains within the research field.

The first cluster (red) was the largest, comprising 88 sources, indicating a highly interconnected body of literature focused primarily on plant pathology and disease management. Within this cluster, *Phytopathology* emerged as the most influential journal, recording 103 links, a total link strength (TLS) of 6958, and 310 citations. Its high connectivity demonstrates its central role in integrating research on plant diseases, host–pathogen interactions, epidemiology, and disease control strategies.

The second cluster (green) consisted of 12 sources and mainly represented journals specializing in fungal biodiversity, taxonomy, and molecular systematics. *Fungal Diversity* was the dominant journal in this cluster, with 58 links, a remarkably high total link strength of 43, 284, and 188 citations. The exceptionally high TLS reflects the journal’s strong intellectual connections with numerous influential sources, emphasizing its importance in studies of fungal phylogeny, biodiversity, and phytopathogenic fungi.

The third cluster (blue) contained 8 sources and represented a more specialized research domain. *Mycosphere* occupied the central position in this cluster, with 50 links, a total link strength of 34, 799, and 150 citations. These findings indicate that *Mycosphere* serves as a major publication outlet for research on fungal taxonomy, ecology, molecular identification, and emerging phytopathogenic species, complementing the broader plant pathology literature represented by the other clusters.

Phytopathology serves as the primary knowledge base for plant pathology, whereas *Fungal Diversity* and *Mycosphere* provide complementary expertise in fungal biodiversity, taxonomy, and molecular characterization. The strong co-citation relationships among these journals demonstrate the growing integration of classical phytopathology with modern mycological and molecular research approaches.

### 3.2. Geographic and Institutional Distribution

**Figure 4:**
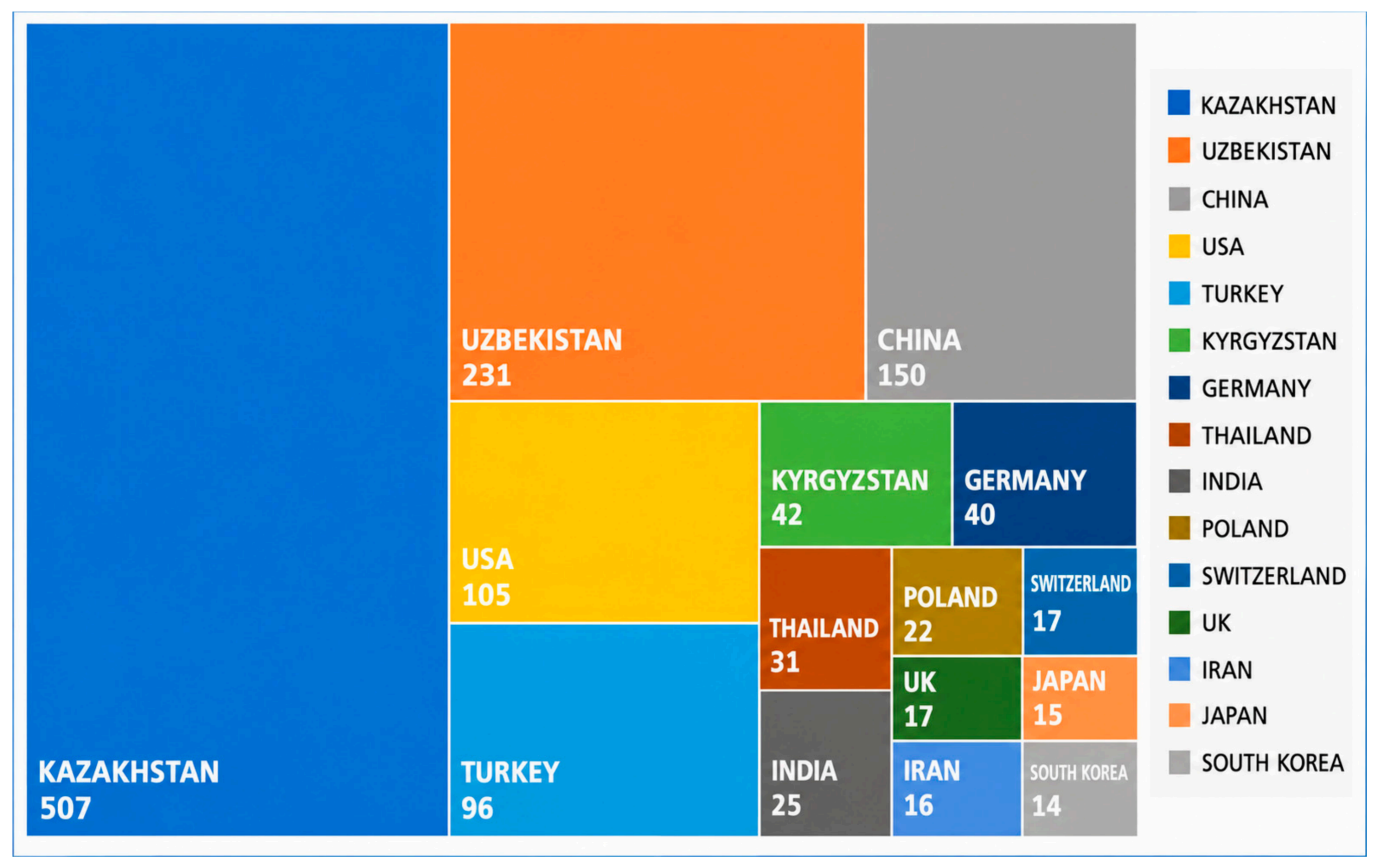
Frequency of country occurrences in the analyzed publications.

The number of publications produced by researchers affiliated with Central Asian institutions and the frequency of country occurrences in author affiliations are widely recognized as important indicators for assessing the geographical distribution and research activity of countries in bibliometric studies. Figure 4 presents the frequency of country occurrences in the analyzed dataset.

The treemap illustrates the relative frequency with which countries appear in the affiliations of the analyzed documents. Larger rectangles indicate higher occurrence frequencies. Kazakhstan exhibited the highest occurrence frequency (507), followed by Uzbekistan (231), China (150), the United States (105), and Türkiye (96). These values represent the frequency with which countries appear in author affiliations. Kazakhstan and Uzbekistan exhibited the highest occurrence frequencies within the Scopus-indexed dataset, reflecting their prominent representation in the analyzed literature.

It should be noted that the country frequencies presented in Figure 4 represent the number of country occurrences in the affiliations of the analyzed publications rather than the number of unique publications. Since a single publication may involve authors from multiple countries, each participating country is counted separately. Consequently, the occurrence frequency of a country may exceed the total number of analyzed publications. High occurrence frequencies therefore indicate stronger representation and participation in the scientific literature rather than a greater number of unique publications.

It should be noted that the country frequencies presented in Figure 4 represent the number of country occurrences in the affiliations of the analyzed publications rather than the number of unique publications. Since a single publication may involve authors from multiple countries, each participating country is counted separately.

Consequently, the occurrence frequency of a country may exceed the total number of analyzed publications. High occurrence frequencies therefore indicate stronger representation and participation in the scientific literature rather than a greater number of unique publications.

**Figure 5:**
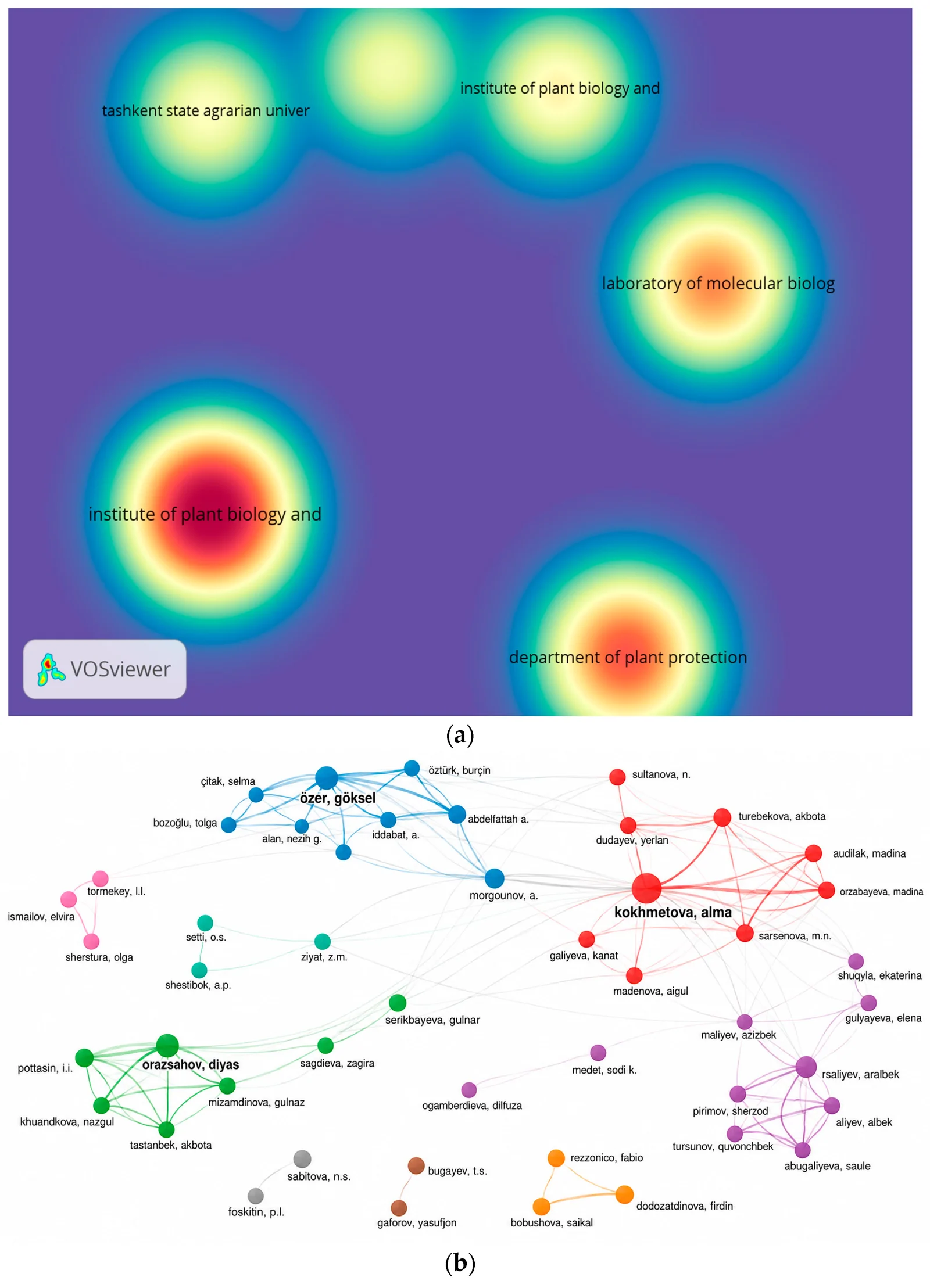
Collaboration analysis of institutions and authors in plant disease. (**a**) Density map of institutional collaboration based on standardized institution names generated by VOSViewer (2023, Leiden, The Netherlands, version 1.6.20). Colors represent the density of institutions and their associated links. Yellow and red areas indicate higher density, whereas blue and purple areas indicate lower density (**b**) Author collaboration network generated using Biblioshiny (2026, Naples, Italy, version 4.6.0). Different colors represent distinct co-authorship clusters, indicating groups of authors connected through collaborative publications.

Figure 5a shows the density distribution of institutional research activity. Based on the selected threshold of at least 5 documents and 4 citations per organization, 13 organizations were identified, forming 6 clusters with 9 collaboration links and a total link strength of 27. The strongest collaboration links were observed among the departments of plant protection, the Institute of Plant Biology and Biotechnology, and the Kazakh National Agrarian Research University. The highest total link strength was recorded for the leading plant protection departments (9 and 8), followed by other plant protection units and the Institute of Plant Biology and Biotechnology (7 each). The visualization suggests that research activity is concentrated around a limited number of organizations, while other institutions occupy less prominent positions in the network.

Figure 5b presents the co-authorship network of researchers in the analyzed field. A total of 49 authors were grouped into 9 collaboration clusters, indicating the presence of several interconnected research groups. Kokhmetova, Alma showed the highest betweenness centrality (290.97), followed by Rsaliyev, Shynbolat (131.97), Dutbayev, Yerlan (131.38), Sapakhova, Zagipa (111.51), and Morgounov, A. (99.19). These authors occupied relatively central positions in the network and served as important links between different co-authorship groups. Other notable contributors included Rsaliyev, Aralbek, Ziyaev, Z.M., Temreshev, I.I., and Özer, Göksel. The network indicates a multi-cluster structure with several key authors connecting otherwise distinct collaboration groups.

Table 3 summarizes the publication performance of different countries based on Single-Country Publications (SCP) and Multiple-Country Publications (MCP) metrics. Among the top 10 productive countries, Kazakhstan produced 143 publications, accounting for 28.31% of the total output, while Uzbekistan contributed 82 publications, representing 16.23%. Both countries demonstrated strong SCP values, with Uzbekistan recording 82 SCPs and Kazakhstan 143 SCPs. However, differences were observed in international collaboration rates, as reflected by MCP%. Uzbekistan achieved an MCP rate of 15.85% (13 publications), whereas Kazakhstan recorded 20.27% (29 publications). In contrast, countries such as China, Turkey, Germany, Korea, and Poland produced relatively fewer publications but exhibited higher MCP percentages, indicating stronger international research collaboration. Meanwhile, countries including the USA and Kyrgyzstan showed comparatively lower MCP-to-SCP ratios, suggesting a greater emphasis on independent national research activities.

To complement the MCP (%) indicator, Total Link Strength (TLS) was used to assess the intensity of international co-authorship. Kazakhstan showed the highest collaboration intensity (TLS—76), followed by Uzbekistan (TLS—54), Türkiye (TLS—53), and the USA (TLS—52). The proportion of internationally collaborative publications (MCP%) and the intensity of collaboration (TLS) provide complementary information on the international research connectivity of individual countries.

### 3.3. Prolific Authors and Research Collaborations

Central Asian plant pathology research is characterized by strong national concentration in Kazakhstan and Uzbekistan, whereas several external countries participate primarily through internationally collaborative publications.

**Figure 6:**
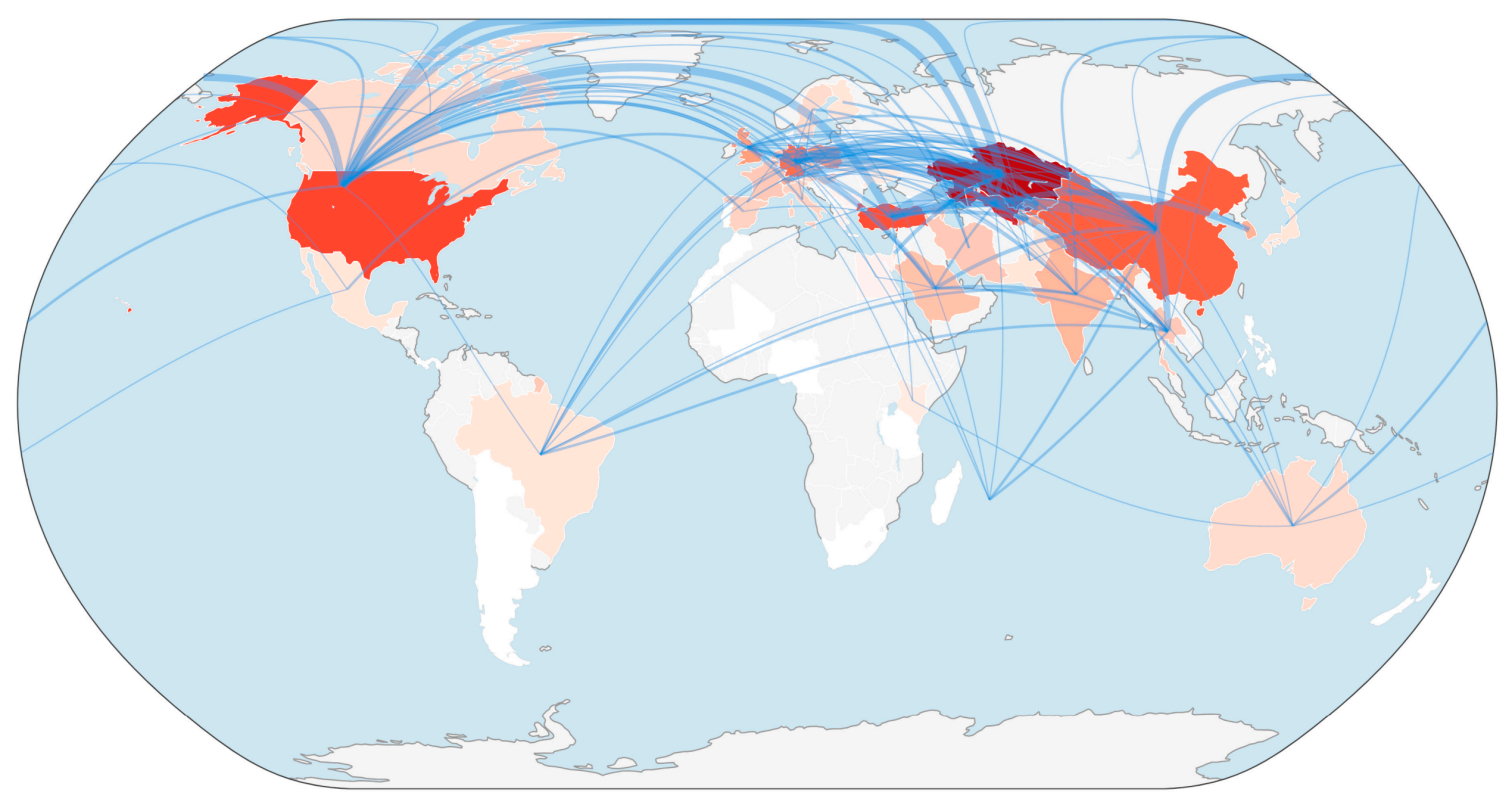
International collaboration world map in the Scopus-indexed dataset, generated using Biblioshiny (2026, Naples, Italy, version 4.6.0). Red-shaded areas represent countries included in the collaboration network, while the blue curved lines indicate international collaboration links between countries.

Figure 6 illustrates the geographical distribution and international collaboration network of research across countries. The collaboration network included 62 countries and 487 inter-country collaboration links, indicating a broad but uneven pattern of international cooperation. Kazakhstan, the USA, Uzbekistan, and China showed the highest collaboration frequencies, with 101, 100, 99, and 97 links, respectively, followed by Germany (82), Türkiye (72), and the United Kingdom (61).

The network centrality analysis further identified China as the most prominent intermediary country, with the highest betweenness centrality (0.159), followed by Switzerland (0.117), Uzbekistan (0.109), Kazakhstan (0.109), and the USA (0.108). These findings indicate that these countries played important roles not only in maintaining extensive collaborative links but also in connecting different parts of the international research network. Overall, the map demonstrates a geographically diverse collaboration structure, although collaboration intensity remains concentrated among a limited number of highly connected countries. 

**Figure 7:**
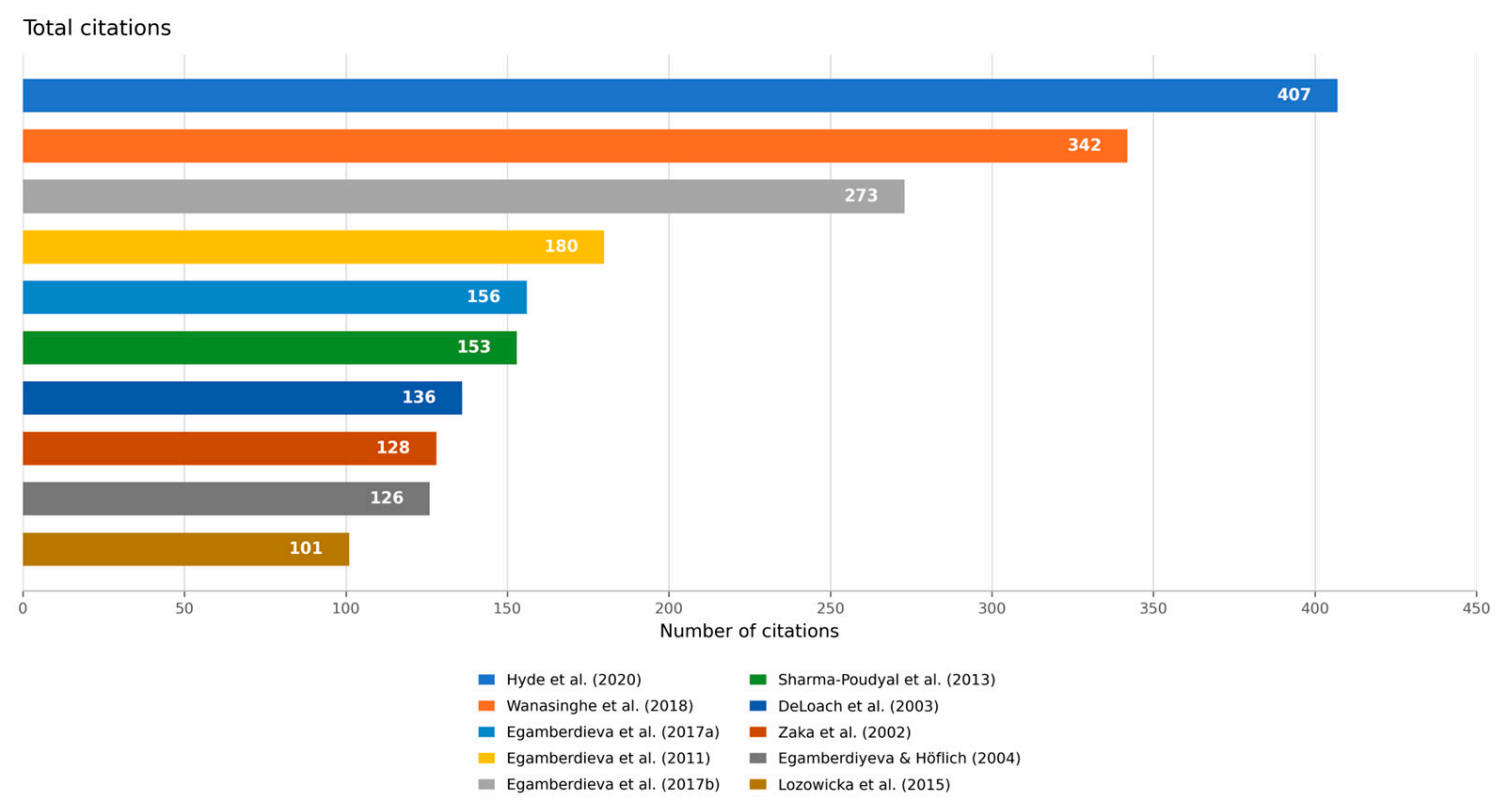
Most cited documents on plant diseases and phytopathogenic threats. Data from [26,27,28,29,30,31,32,33,34,35].

In bibliometric studies, the number of publications and citation counts associated with individual authors are commonly used to identify the most productive, influential, and active researchers within a particular field. Figure 7 presents the most globally cited publications related to research in plant diseases and phytopathogenic threats. Among these, the study conducted by Hyde KD achieved the highest total citations (TC—407), establishing the authors among the most influential contributors in this research domain [26]. The study is devoted to fungal taxonomy and biodiversity, focusing on the discovery, identification, and classification of novel fungal taxa using morphological and molecular phylogenetic approaches. It provides a comprehensive global update on fungal diversity by describing numerous new taxa and reporting new host associations and geographical distributions, thereby contributing to a better understanding of fungal evolution and biodiversity.

Wanasinghe et al. (2018) ranked second with 342 total citations, while Egamberdieva et al. (2017) occupied the third position with 273 citations [27,28]. Wanasinghe et al. (2018) [27] focused on the taxonomy and biodiversity of fungi associated primarily with Rosa species. Using integrated morphological and molecular phylogenetic analyses, the study described numerous new fungal genera and species, expanded knowledge of fungal diversity, and provided new host and distribution records, thereby contributing to a better understanding of fungal evolution and taxonomy. Additional influential contributions were made by Egamberdieva et al. [29], Egamberdieva et al. [30], and Sharma-Poudyal et al. [31], with total citation counts of 227, 180, 156, and 153, respectively. Egamberdieva et al. (2011) [29] focus on the use of plant growth-promoting rhizobacteria (PGPR) to suppress *Fusarium solani* and improve cucumber growth and yield under saline soil conditions through microbial seed priming. Egamberdieva et al. (2017) [28] investigated endophytic bacteria associated with medicinal plants in Uzbekistan and evaluated their potential as biological control agents and plant growth-promoting microorganisms against plant pathogens. Sharma-Poudyal et al. (2013) investigated the global virulence diversity of *Puccinia striiformis* f. sp. *Tritici*, identifying virulence patterns and resistance gene effectiveness to support wheat breeding and stripe rust management [31].

As shown in Table 4, author contributions were concentrated among a relatively small group of productive researchers. Kokhmetova, Alma ranked first in publication output with 22 articles, while Morgounov, A. received 355 citations from 18 publications. Egamberdieva, Dilfuza demonstrated the highest citation impact, with 827 citations across 14 publications, indicating substantial scholarly influence despite a moderate publication output. The leading authors were predominantly affiliated with Kazakhstan, which accounted for six of the top ten researchers, followed by Türkiye, Uzbekistan, and Kyrgyzstan.

**Figure 8:**
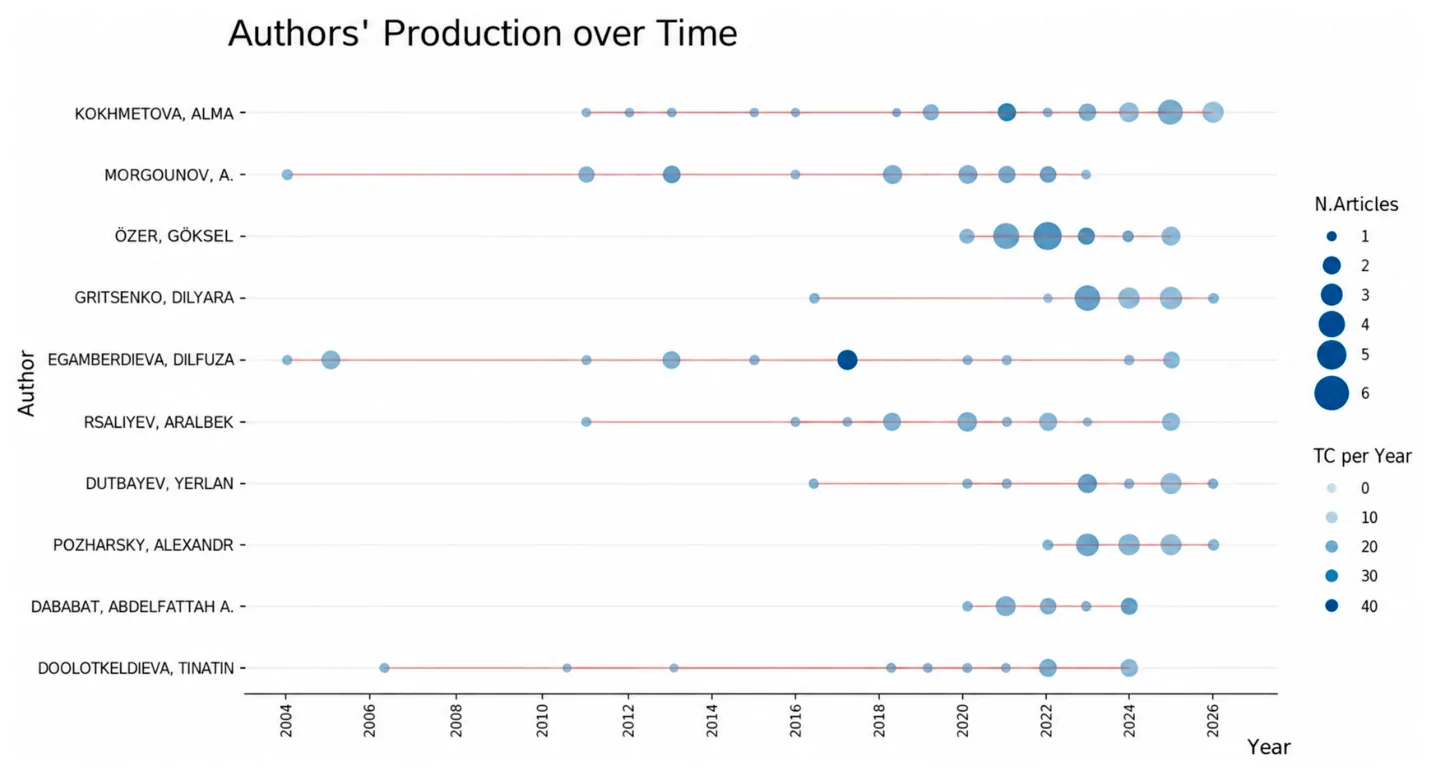
Author production over time. The horizontal red lines indicate the period of the author’s publication activity.

Figure 8 shows the temporal distribution of publications by the leading authors. Kokhmetova, Alma demonstrated the longest and most consistent publication activity, with studies spanning 2011–2026. Other leading authors, including Morgounov, A., Özer, Göksel, and Egamberdieva, Dilfuza, also showed sustained contributions, particularly during 2021–2025. Author productivity increased mainly in recent years, with research focusing predominantly on wheat diseases, pathogen resistance, root rot, and plant protection.

Table 5 presents the co-authorship profile of the leading researchers. Gritsenko, D. showed the highest total link strength (TLS—35) and the largest publication output (14 articles), followed by Kokhmetova, A. (TLS—26; 13 publications). Pozharskiy, A. and Nizamdinova, G. both had a TLS of 22, with 10 and 9 publications, respectively. Rsaliyev, A. and Rsaliyev, Sh. Belonged to Cluster 3, while the remaining authors were mainly distributed between Clusters 1 and 2. Overall, the results indicate that Gritsenko and Kokhmetova were the most strongly connected authors, whereas the co-authorship network consisted of three main collaboration clusters.

### 3.4. Intellectual Structure and Bibliographic Coupling

Science mapping techniques such as historiograph, and bibliographic coupling are widely applied to explore relationships among research publications and to identify dominant research themes. 

**Figure 9:**
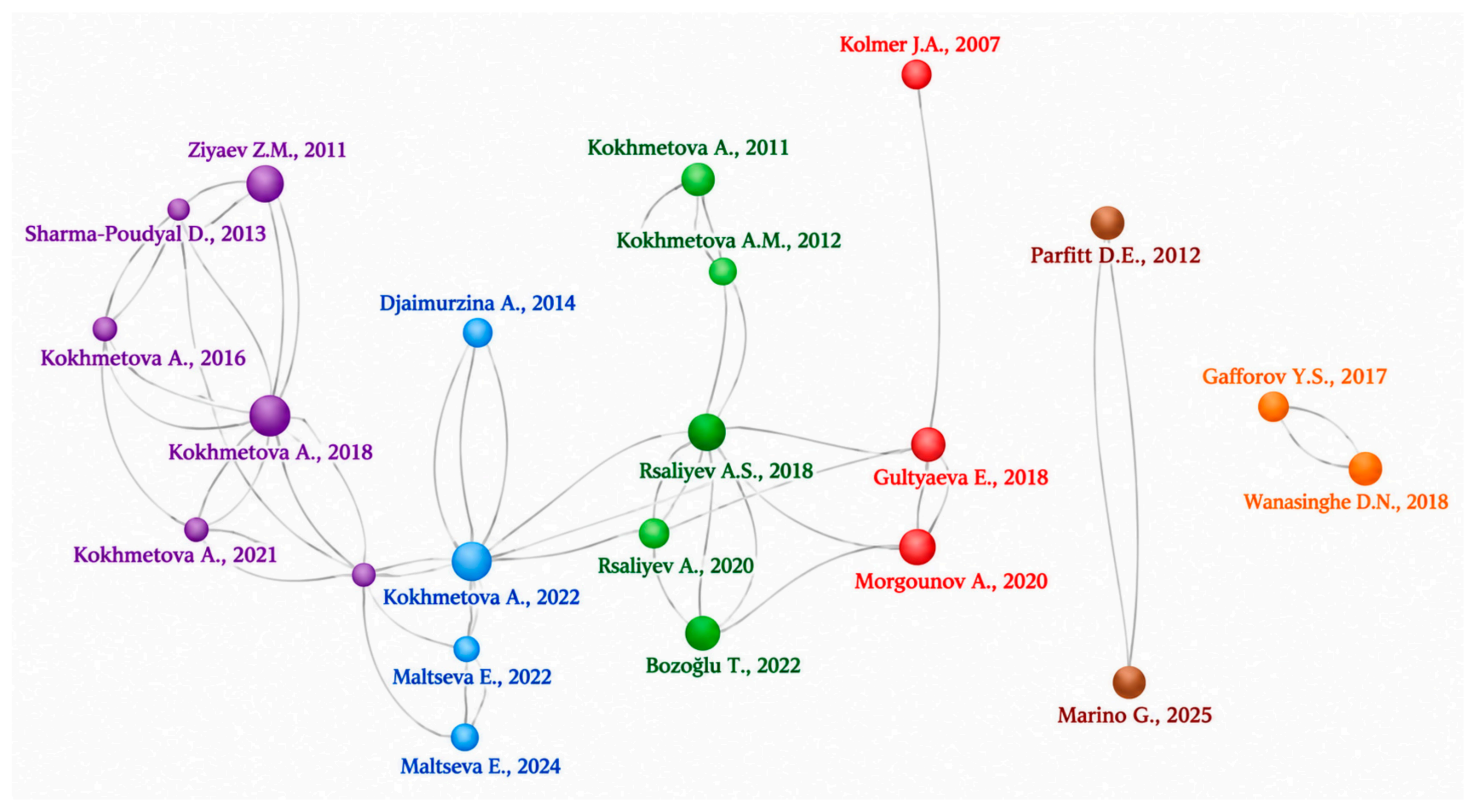
Historiograph networks of research on plant diseases and phytopathogenic threats in Central Asia. Data from [6,27,31,37,41,44,45,46,47,48,49,50,51,52,53,54,55,56,57,58,59].

Figure 9 presents the historiograph network of key publications and their citation relationships within the analyzed research field. The network comprises six thematic clusters, with the largest clusters focused on wheat rust diseases, disease resistance, and *Erwinia amylovora*-associated fire blight. The central clusters demonstrate strong connections among studies on wheat resistance, pathogen virulence, race structure, and molecular characterization, particularly in Central Asia and Kazakhstan. A separate cluster represents research on fire blight and *E. amylovora*, while smaller clusters concern pistachio research and fungal taxonomy and phylogeny. Overall, the historiograph highlights the development of research from pathogen identification and disease characterization toward molecular, genetic, and resistance-oriented studies. 

In particular, bibliographic coupling assumes that publications sharing similar references are likely to address related topics or methodologies. Based on shared citations, publications can therefore be grouped into thematic clusters that reflect common research interests.

**Figure 10:**
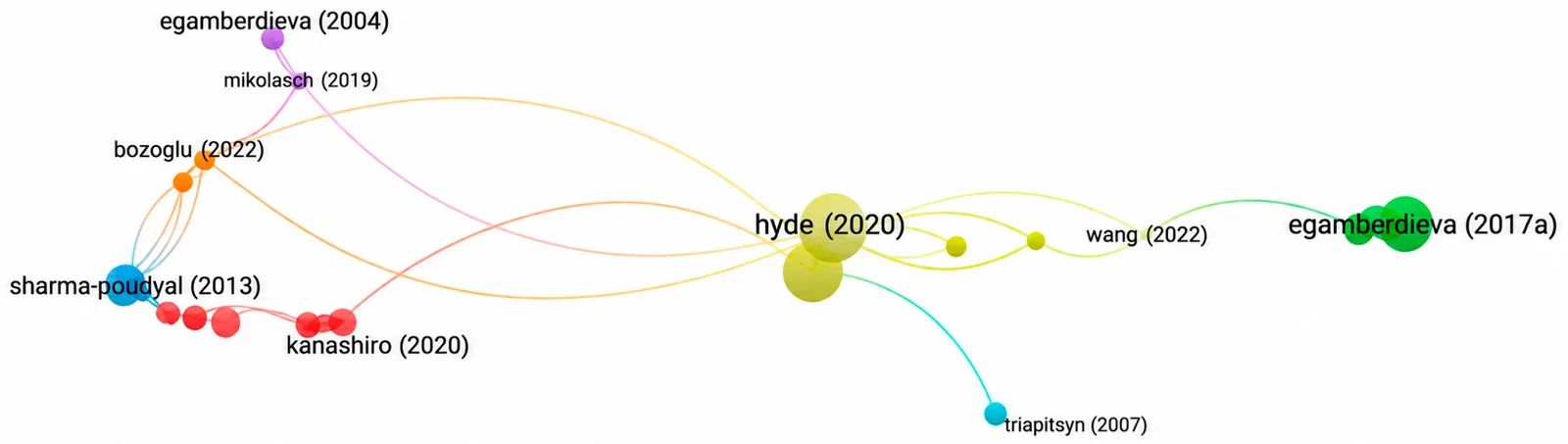
Bibliographic coupling network of publications on plant diseases and phytopathogenic threats generated by VOSViewer (2023, Leiden, The Netherlands, version 1.6.20). Data from [26,28,31,34,60,61,62,63,64].

Figure 10 presents the bibliographic coupling network of studies related to plant disease management. In the VOSviewer analysis, the minimum citation threshold was set to 30. The results identified seven major thematic clusters, including cluster 1 (red) with 8 publications, cluster 2 (green) with 6 publications, cluster 3 (blue) with 3 publications, cluster 4 (yellow) with 5 publications, cluster 5 (purple) with 3 publications, cluster 6 (light blue) with 2 publications, and cluster 7 (orange) with 2 publications.

The first cluster is primarily focused on apple diseases, host–pathogen interactions, pathogen population genetics, and sustainable disease management. The most influential publications within this cluster are those by Gladieux et al. (2010), Kanashiro et al. (2020), and Jurick et al. (2011), which recorded Total Link Strength (TLS) values of 7, 3, and 3, respectively, indicating strong thematic relationships among these studies [60,65,66]. These highly connected studies illustrate the integration of pathogen evolution, host resistance, and sustainable disease management strategies in fruit crop protection.

The second cluster is centered on plant growth-promoting rhizobacteria (PGPR), biological control, and sustainable crop production under saline conditions. The most influential publications in this cluster were authored by Egamberdieva et al. (2011) and Egamberdieva et al. (2017), with Total Link Strength (TLS) values of 18 and 14, respectively, reflecting their strong influence on this research theme [28,29]. These studies demonstrated the effectiveness of salt-tolerant beneficial bacterial strains, including Pseudomonas, Serratia, and Stenotrophomonas, in suppressing *Fusarium solani*, reducing disease incidence, and enhancing cucumber growth and fruit yield in saline soils. Their findings highlight the potential of microbial seed priming as an environmentally friendly strategy to improve crop productivity and disease resistance under increasing soil salinity, particularly in the agricultural conditions of Uzbekistan.

The third cluster is primarily associated with wheat stripe rust, pathogen virulence, resistance genes, and disease management in Central Asia. The most influential studies in this cluster were published by Sharma-Poudyal et al. (2013), Kokhmetova et al. (2021), and Sharma et al. (2016), with Total Link Strength (TLS) values of 19, 13, and 4, respectively, indicating their central role in this research area [31,41,67]. Collectively, these publications emphasize the integration of pathogen surveillance, marker-assisted breeding, and effective disease management practices to enhance durable wheat resistance against stripe rust.

The fourth cluster primarily focuses on fungal taxonomy, molecular phylogenetics, and fungal biodiversity, with particular emphasis on the discovery and classification of novel fungal taxa. The most influential studies in this cluster were conducted by Hyde et al. (2020), Wanasinghe et al. (2018), and Gafforov et al. (2017), with Total Link Strength (TLS) values of 75, 77, and 6, respectively, indicating their strong interconnectedness within this research domain [26,27,54]. These studies demonstrate the growing importance of molecular taxonomy and biodiversity surveys for understanding fungal diversity and supporting plant disease research.

The fifth cluster is primarily related to plant growth-promoting microorganisms, soil microbiology, and environmental bioremediation. The most influential studies in this cluster were published by Egamberdieva et al. (2004) and Mikolasch et al. (2019), with Total Link Strength (TLS) values of 11 and 4, respectively [34,64]. Together, these studies highlight the dual role of beneficial microorganisms in promoting sustainable agriculture and supporting the bioremediation of contaminated environments.

These clusters encompass fruit crop diseases and host-pathogen interactions, plant growth-promoting microorganisms and biological control, wheat stripe rust resistance and disease management, fungal taxonomy and biodiversity, and microbial applications in sustainable agriculture and environmental bioremediation. Together, these interconnected research themes demonstrate a growing emphasis on integrating molecular approaches, microbial technologies, and sustainable management strategies to improve crop health, strengthen disease resistance, and support environmentally sustainable agricultural systems.

### 3.5. Conceptual Structure and Keyword Analysis

**Figure 11:**
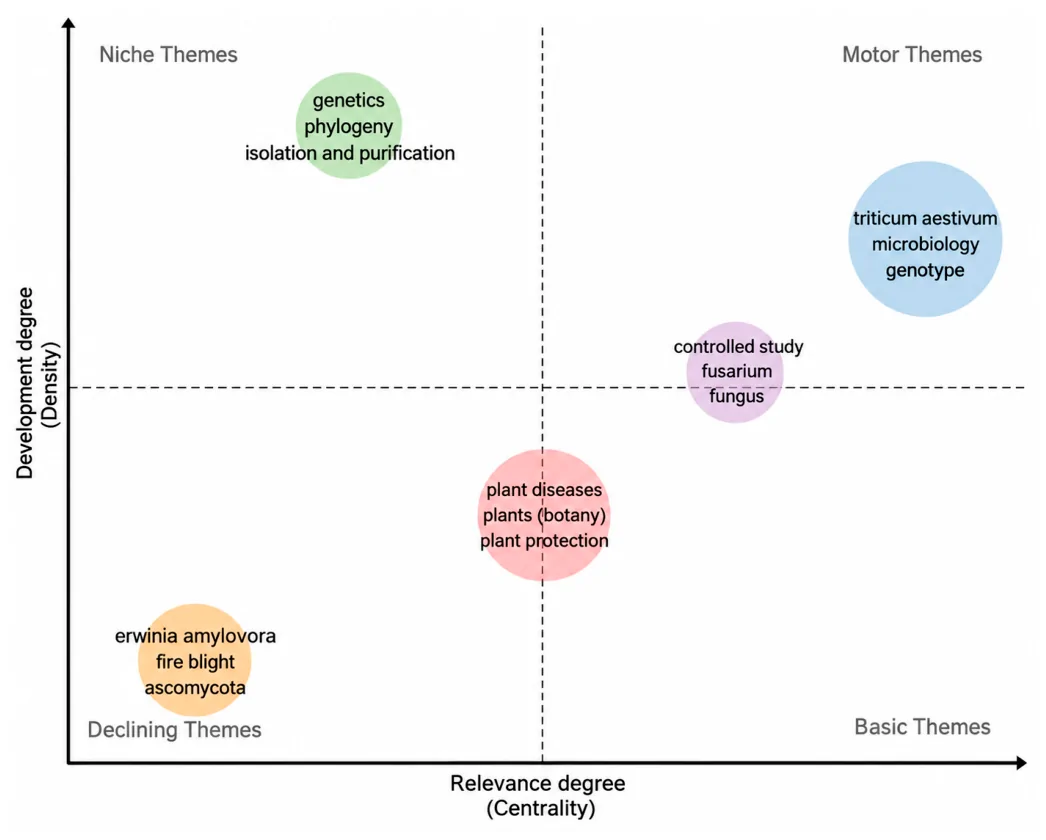
Visualization of thematic map for dominant themes in plant disease and phytopathogenic threats for 1994–2026. The red cluster, comprising plant diseases, plants (botany), and plant protection, is located in the lower-left quadrant (Declining Themes). Purple cluster is located near the boundary between the Motor Themes and Basic Themes quadrants, predominantly within the Motor Themes quadrant.

The thematic map based on co-word analysis illustrates the conceptual structure and development of the research field (Figure 11). The thematic map revealed five major thematic groups distributed across four conceptual quadrants according to their centrality and density. The motor themes quadrant contained *Triticum aestivum*, microbiology, and genotype, indicating a well-developed and highly relevant research direction. The controlled study, *Fusarium*, and fungus cluster was positioned close to the boundary between motor and basic themes, suggesting substantial relevance but comparatively moderate thematic development.

The niche themes quadrant included genetics, phylogeny, and isolation and purification. These topics showed relatively high development but lower centrality, indicating specialized research areas with limited integration into the broader thematic structure. In contrast, plant diseases, plants (botany), and plant protection were located in the basic theme quadrant, reflecting foundational topics with relatively high relevance but lower development.

The emerging themes quadrant contained *Erwinia amylovora*, fire blight, Ascomycota, and declining themes. Their position suggests comparatively low centrality and development within the overall research structure. 

**Figure 12:**
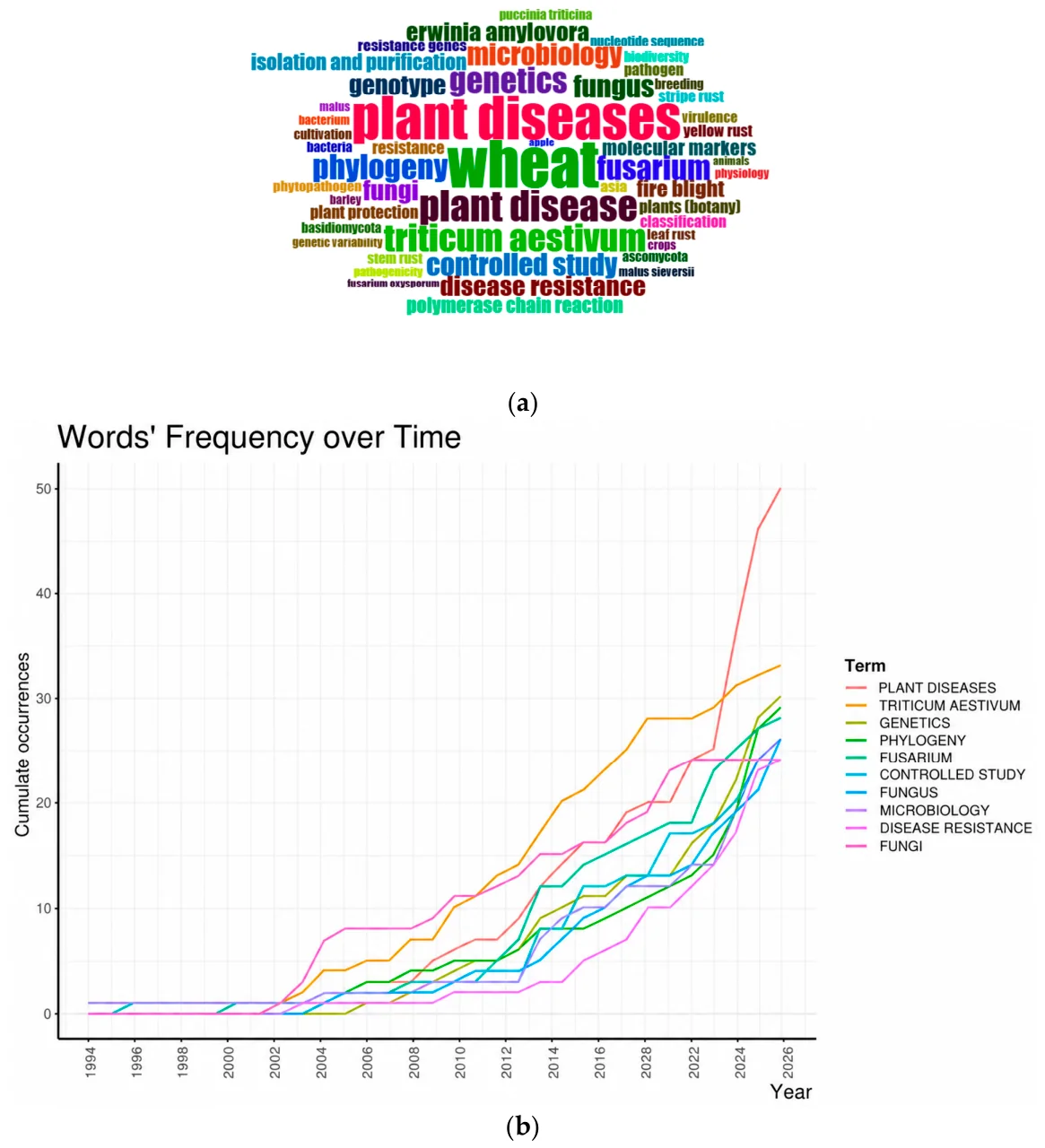
Keyword distribution and temporal trends in the analyzed literature: (**a**) Keyword cloud illustrating the most frequently occurring terms in the analyzed literature; (**b**) Temporal trends in the frequency of the most occurring keywords.

The word cloud shown in Figure 12a provides an overview of the predominant themes and research directions identified in the analyzed literature. The frequency analysis revealed that Central Asian plant disease research is strongly focused on wheat, plant diseases, phytopathogens, and disease resistance. “Wheat” was the most frequent term (55 occurrences), followed by “plant diseases” (50), “plant disease” (36), and *Triticum aestivum* (33). Genetic and molecular research was also prominent, as reflected by “genetics” (30), “phylogeny” (29), “genotype” (23), “molecular markers” (18), and “polymerase chain reaction” (18).

Fungal pathogens represented another major research focus, particularly *Fusarium* (28), “fungus” (26), and “fungi” (24), while bacterial disease research was strongly represented by *Erwinia amylovora* (22) and “fire blight” (20). Disease resistance was also highly prominent, with “disease resistance” occurring 24 times and “stripe rust,” “yellow rust,” “leaf rust,” and “stem rust” occurring 14, 14, 13, and 13 times, respectively. Overall, the frequency patterns indicate a strong emphasis on cereal disease resistance, pathogen characterization, molecular genetics, and disease management, reflecting the predominantly agricultural and molecular orientation of plant disease research in Central Asia.

Figure 12b illustrates the temporal dynamics of the most frequently occurring keywords from 1994 to 2026. The results show a marked increase in keyword frequency, particularly during the most recent years. Plant diseases were consistently present throughout the study period and increased from 1 occurrence in 1994 to 50 in 2026, indicating its persistent importance in the regional research landscape. *Triticum aestivum* also showed substantial growth, particularly after 2010, reaching 33 occurrences in 2026.

Other major terms displayed a marked increase after 2014–2015. *Fusarium* increased from 1 occurrence in 2001 to 28 in 2026, while fungi increased to 24 and fungus to 26. Similarly, genetics and phylogeny increased to 30 and 29 occurrences, respectively. Disease resistance appeared in 2004 and increased to 24 occurrences by 2026.

The strongest expansion occurred during 2015–2026, when most of the analyzed terms increased substantially, suggesting a growing diversification and molecular orientation of plant disease research in Central Asia.

**Figure 13:**
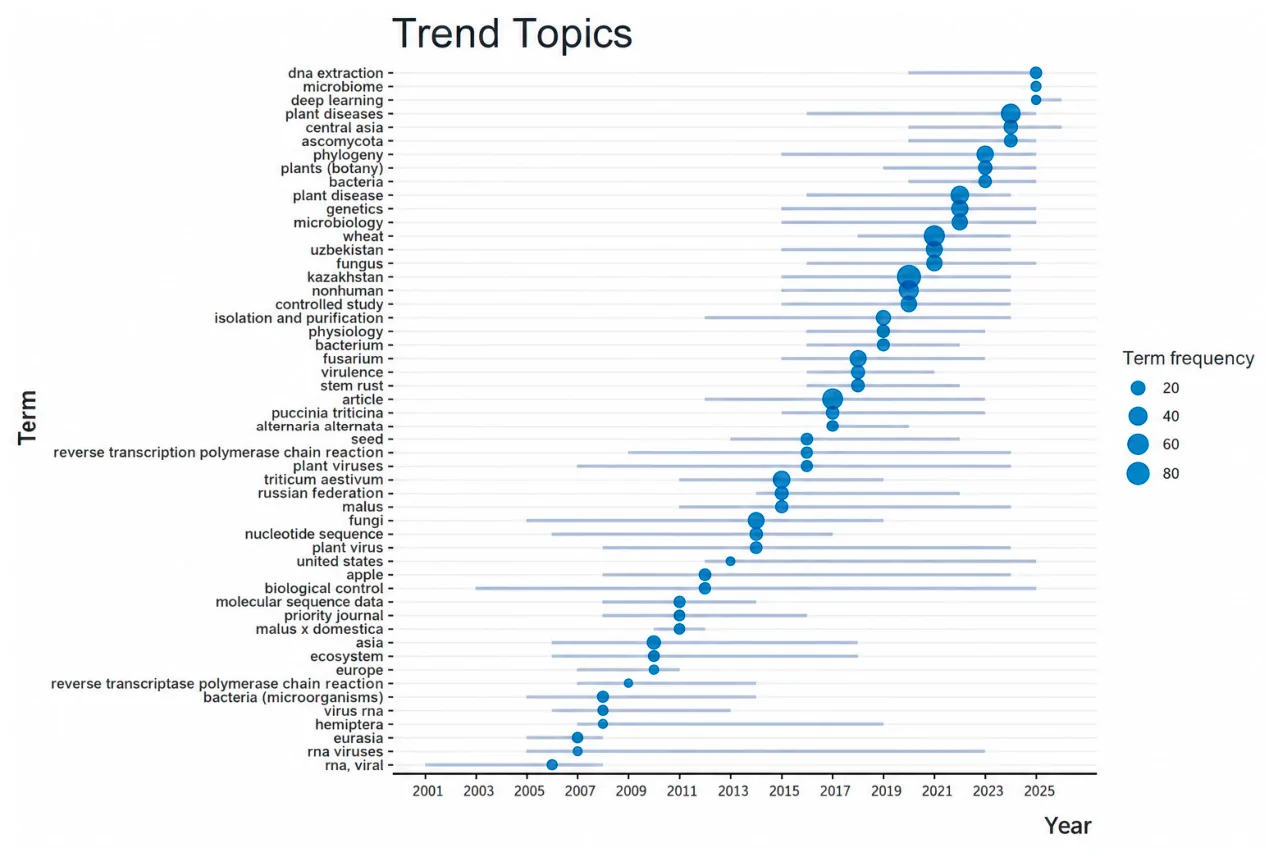
Trend topics and their temporal evolution in the analyzed literature. Horizontal lines represent the time span of keyword occurrence in the analyzed publications.

The trend-topic analysis reveals a clear shift in research focus over time. Early studies were mainly associated with RNA viruses, bacteria, molecular sequence data, apple, and ecosystem-related topics. During 2014–2019, research increasingly focused on fungi, *Triticum aestivum*, plant viruses, *Fusarium*, virulence, and wheat diseases. More recent research has shifted toward plant diseases, genetics, microbiology, phylogeny, Central Asia, and DNA extraction, while microbiome and deep learning represent emerging topics in 2025–2026. Figure 13 indicates a transition from conventional pathogen identification toward molecular, genetic, phylogenetic, and advanced analytical approaches.

**Figure 14:**
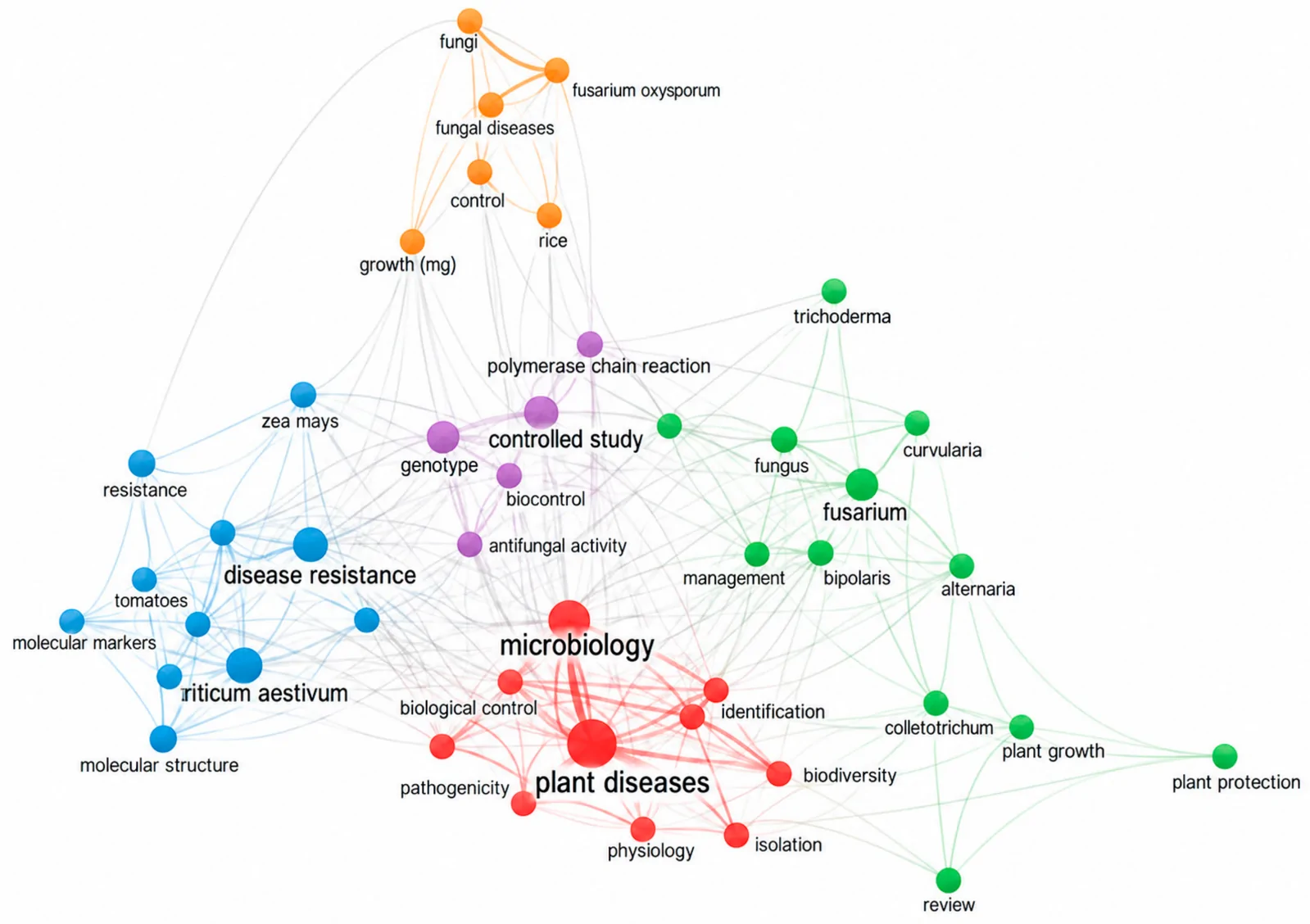
Keyword co-occurrence network showing the major thematic clusters and their interconnections in plant disease research. Red: plant pathogens, plant protection, and disease management; blue: wheat diseases, host resistance, and breeding; green: plant disease microbiology, genetics, phylogeny, and pathogen identification; purple: biocontrol, controlled study and genotype; yellow:fungi, fungal diseases and growth.

Co-word analysis is an effective bibliometric approach for identifying and visualizing the major research themes and conceptual relationships within a body of scholarly literature through the co-occurrence of keywords [68]. To further elucidate the conceptual structure of the research field and identify the major thematic areas, a keyword co-occurrence network was constructed (Figure 14). The co-word network analysis identified five thematic clusters reflecting the major research directions in plant disease and phytopathogen research in Central Asia. Cluster 1 was centered on plant diseases, genetics, microbiology, and phylogeny. Plant diseases showed the highest betweenness centrality (229.56) and PageRank (0.0697), indicating its central position within the overall research network, while genetics (98.53) and microbiology (71.48) also played important connecting roles.

Cluster 2 focused mainly on wheat disease resistance and rust-related research, including *Triticum aestivum*, disease resistance, resistance genes, stripe rust, yellow rust, leaf rust, and stem rust. *Triticum aestivum* and disease resistance showed relatively high betweenness values of 69.13 and 67.86, respectively. Cluster 3 represented fungal and phytopathogen research, with *Fusarium*, fungus, fungi, phytopathogen, bacteria, and *Fusarium oxysporum* as prominent terms.

Cluster 4 reflected molecular and genetic characterization, particularly polymerase chain reaction (betweenness = 56.11), controlled study, genotype, nucleotide sequence, and genetic variability. Cluster 5 was associated with bacterial fire blight research and apple hosts, including *Erwinia amylovora*, fire blight, *Malus sieversii*, apple, and genotyping. Among these terms, genotyping showed the highest betweenness (33.62).

Overall, the network demonstrates that research in the region is structured around plant disease biology, wheat disease resistance, fungal and bacterial pathogens, and molecular/genetic characterization, with plant diseases, genetics, microbiology, and disease resistance serving as important connecting themes.

An important feature of the network is the strong inter-cluster connectivity, represented by the numerous links connecting the red, blue, and green groups. These connections indicate that research on plant pathogens and plant protection is not isolated from studies of wheat disease resistance or molecular and phylogenetic characterization. In particular, the links between pathogen-related terms in the red cluster and genetics, microbiology and phylogeny in the green cluster suggest an increasing integration of pathogen identification and characterization with molecular and genetic approaches. Similarly, the connections between the blue cluster and the other clusters indicate that studies of wheat diseases increasingly incorporate pathogen biology, resistance genetics, and molecular characterization.

## 4. Discussions

### 4.1. Evolution of Plant Disease Research in Central Asia

The evolution of plant disease research in Central Asia over the past three decades shows a gradual shift from traditional pathogen identification and disease characterization to molecular characterization, host resistance genetics, biological control, and more recently genomic and data-driven approaches. The earliest publications identified in the dataset were primarily concerned with the occurrence, identification, and characterization of plant viruses and disease-associated microorganisms. For example, Spak and Novikov (1994) reported mixed infections of turnip mosaic virus, cauliflower mosaic virus, and cucumber mosaic virus in winter oilseed rape from Uzbekistan, representing an early stage of pathogen surveillance in the region [69]. Similarly, molecular analysis of beet necrotic yellow vein virus subsequently led to the identification of a unique virus type in Kazakhstan, marking an early shift in the molecular characterization of plant viruses [70]. These studies demonstrate that the initial phase of the regional research landscape was largely focused on documenting pathogen occurrences and identifying and determining the distribution of economically important pathogens. In the early 2000s, disease resistance research became increasingly linked to the characterization and utilization of Central Asian plant genetic resources. This was particularly evident in apple and wheat research. Wild *Malus* populations originating in Central Asia were evaluated for fire resistance, highlighting the importance of the region as a source of disease resistance genetic material [71]. Subsequent studies of *Malus sieversii* populations from Kazakhstan further highlighted their potential for disease resistance and horticultural improvement [72]. In cereals, Singrün et al. studied genes for resistance to powdery mildew and leaf rust in wheat varieties originating from the Caucasus, Central Asia and Inner Asia [73]. Thus, plant disease research during this period moved beyond the emergence of pathogens to the use of regional germplasm to identify host resistance and improve yields.

A parallel research focus has emerged around beneficial microorganisms and biological approaches to crop protection, particularly in Uzbekistan. Egamberdiyeva and Höflich studied plant growth-promoting bacteria in cotton and chickpea grown under semi-arid conditions [34]. Although these studies were not exclusively focused on plant diseases, they provided an important biological basis for further research on microbial disease control. This direction became more clearly linked to plant disease management in subsequent studies, particularly through the use of beneficial rhizobacteria and endophytic microorganisms as biological control agents.

Since about 2010, wheat diseases, particularly rusts and host resistance, have increasingly dominated the research area. Screening of wheat germplasm for rust resistance has begun to incorporate both conventional and molecular methods, indicating a clear methodological shift in disease resistance research [55]. This trend has been reinforced by studies identifying molecular markers associated with resistance to wheat stem and leaf rust [56,59]. Overall, these studies demonstrate that Central Asian plant pathology has increasingly incorporated pathogen population biology and molecular genetics alongside traditional phenotypic disease assessment. 

The prominence of wheat disease resistance and molecular breeding in the identified research landscape is consistent with the documented importance of wheat diseases in Central Asian agriculture. Wheat rusts, particularly stripe rust and leaf rust, have received considerable attention because of their potential to cause substantial yield losses. Studies evaluating Central Asian wheat germplasm have identified sources of resistance and demonstrated the importance of resistance genes and molecular markers in breeding programs [7,37,41]. At the same time, research on pathogen virulence has emphasized the need for continuous monitoring of pathogen populations because changes in virulence can reduce the effectiveness of previously deployed resistance genes [31,42,43]. Beyond host resistance, research in the region has also investigated biological approaches to disease suppression. Plant growth-promoting rhizobacteria and beneficial endophytic microorganisms have demonstrated potential for suppressing soil-borne pathogens and improving crop performance, particularly under saline and other environmental stress conditions [28,29,30,54,67]. Taken together, these findings indicate that the regional research landscape is increasingly oriented toward integrating host resistance, pathogen monitoring, microbial technologies, and sustainable disease management.

The growing importance of wheat rust research has been particularly evident in Kazakhstan. Turuspekov et al. (2016) used genome-wide association analysis to study stem rust resistance in Kazakh spring barley lines, while Genievskaya et al. (2020) used genome-wide association mapping to characterize resistance to leaf, stem, and yellow rusts in common wheat under field conditions in southern Kazakhstan [74,75]. Similarly, Morgounov et al. (2020) studied the genetic basis of leaf rust resistance in spring wheat in Kazakhstan and Russia [57]. These studies demonstrate a significant shift towards genomics-based disease resistance research, where disease phenotype identification is combined with molecular markers and genome-wide analyses. This development was further strengthened by studies identifying linear rust resistance genes in common wheat varieties and breeding lines in Kazakhstan and later by genome-wide association studies of rust resistance in wheat germplasm collected from Kazakhstan [41,76].

The period after 2020 has been a particularly important one in the development of plant disease research in Central Asia, with a significant expansion from individual pathogen studies to pathogen diversity, disease complexes, and molecular epidemiology. In Kazakhstan, Bozoglu et al. (2022) described fungal pathogens associated with crown and root rot of wheat in the central, eastern, and southeastern regions, while Olivera et al. (2022) demonstrated significant diversity and previously unrecognized virulence pathotypes in populations of the wheat stem rust pathogen responsible for recent outbreaks in Kazakhstan [6,63]. Subsequent studies in Kyrgyzstan identified fungal pathogens associated with crown and root rot in wheat-growing areas in northern Kyrgyzstan [77]. These studies demonstrate that recent research is increasingly focusing on pathogen diversity and disease complexes at the regional scale, rather than at the individual field scale. The second major thematic direction identified in the analysis was related to plant pathogens, fungal biodiversity, and molecular phylogenetics. Highly cited studies in the analyzed literature have focused on fungal taxonomy, biodiversity, and phylogenetic characterization, providing information necessary for identifying new fungal taxa, host associations, and potential phytopathogenic species [26,27,68]. The importance of these approaches is further supported by studies documenting fungal diversity in Central Asia and investigating microorganisms associated with agricultural and contaminated environments [68]. Molecular identification and phylogenetic analysis are therefore increasingly relevant for distinguishing closely related pathogens and improving the taxonomic resolution required for plant disease diagnosis and surveillance.

During the same period, research on biological control and microbial alternatives to traditional chemical disease management has intensified. Egamberdieva et al. (2017) showed that endophytic bacteria can improve pea growth and suppress root rot caused by *Fusarium solani* under salinity stress [30]. In Kazakhstan, Shemshura et al. (2020) studied the antagonistic activity of *Bacillus amyloliquefaciens* MB40 against fire blight, while Doolotkeldieva et al. (2022) evaluated the biological activity of Ag- and Cu-based nanoparticles and their composites against plant pathogens [78,79]. Recent studies have also investigated antagonistic *Trichoderma* species against soil pathogens in Kazakhstan and plant-associated endophytic bacteria in Uzbekistan. These studies reflect a broader shift from pathogen-focused research to integrated and ecologically focused disease management strategies. 

The latest publications also show the emergence of data-rich and AI-driven approaches. For example, the dataset includes studies on tomato disease detection using machine learning and computer vision in 2025, and crop disease detection based on deep learning in 2026. At the same time, microbiome and community-level studies are increasingly gaining prominence, demonstrating the potential for high-throughput characterization of plant-associated microbial communities. However, these topics should be viewed as emerging trends rather than established dominant trends at this time, especially since the publications indexed in 2026 represent only one year of the dataset.

### 4.2. International Collaboration and Regional Research Disparities

The analysis of international collaborations showed that plant disease research in Central Asia is increasingly integrated into the global scientific network, although significant differences between countries remain.

The internationalization of the field is particularly evident in research on emerging pathogens and diseases. For example, research on stripe rust has involved collaboration between Central Asian and international research groups. Baloch et al. investigated the stripe rust resistance gene *Yr15* in wheat germplasm from Turkey and Kazakhstan, with the participation of researchers from several countries [80]. Similarly, Kharipzhanova et al. (2024) investigated *Bipolaris sorokiniana* in wheat and barley in southeastern Kazakhstan through a collaboration involving researchers from Kazakhstan, Kyrgyzstan, Turkey, and the United States [4]. These examples illustrate how research collaborations involving Central Asian countries connect regional crop and pathogen studies with international expertise, particularly in wheat disease resistance, pathogen characterization, and disease management.

International collaboration is also particularly important for the molecular epidemiology of transboundary pathogens. Rezzonico et al. investigated the introduction patterns of fire blight in Central Asia and the Caucasus using CRISPR spacer typing and genome analysis, involving researchers from Kyrgyzstan, Switzerland, and Russia [3]. More recently, Awais et al. investigated the genetic relationship and gene flow of *Puccinia striiformis* f. sp. *tritici* between Uzbekistan and Xinjiang, China [81]. Similarly, Wang et al. demonstrated a close genetic relationship between *Erwinia amylovora* populations in China and populations in Kyrgyzstan and Kazakhstan [82]. These studies highlight the importance of transboundary collaboration for understanding pathogen distribution and epidemiological linkages across Central Asia.

The dataset also shows increasing collaboration in molecular characterization and disease resistance research. For example, Jobe et al. brought together researchers from Uzbekistan and the United States to characterize *Fusarium* isolates from cotton roots [83], while Ochilov et al. collaborated with Chinese researchers to develop bread wheat lines resistant to yellow rust using marker-assisted backcrossing [84]. Such studies demonstrate that international collaboration is increasingly providing access to advanced molecular, genomic, and breeding technologies that complement regional expertise.

The findings suggest that international collaboration represents a significant strength of plant disease research in Central Asia, but research capacity is unevenly distributed across the region. Kazakhstan and Uzbekistan act as key regional contributors, while collaboration with countries such as China, Turkey, Switzerland, Germany, and the United States provides important opportunities for advanced pathogen characterization, molecular breeding, and disease epidemiology. Therefore, strengthening cooperation among the five Central Asian countries, combined with sustained international cooperation, can help reduce regional disparities and support coordinated surveillance and management of transboundary plant pathogens.

### 4.3. Implications for Central Asian Plant Health Policy and Research Priorities

The observed differences in research capacity and collaboration should also be considered within the broader historical and socioeconomic context of Central Asia. The region inherited scientific and agricultural research institutions from the Soviet period, while the post-independence period involved institutional restructuring and changes in research funding and international scientific engagement. Subsequent differences in national investment in research infrastructure, institutional development, international partnerships, and agricultural priorities may have contributed to the uneven development of research capacity observed in the bibliometric landscape. Political and economic factors may also influence researchers’ access to international networks, research infrastructure, funding opportunities, and internationally indexed publication venues. These contextual factors provide plausible explanations for some of the observed differences, although the present bibliometric design does not allow causal effects of individual historical or economic factors to be quantified.

The bibliometric findings have several important implications for plant health policy and research priorities in Central Asia. First, the increasing evidence of recurrent pathogen outbreaks and the variability of pathogen populations suggests the need to strengthen regional surveillance and early warning systems. Wheat stripe rust is a clear example: stripe rust has caused several outbreaks in Central Asia, and recent studies have shown significant changes in pathogen populations. Ziyaev et al. noted the limited information on effective resistance genes, while recent studies have documented the composition and virulence of the *Puccinia striiformis* f. sp. *tritici* population in Kazakhstan [43,58]. In addition, molecular genotyping revealed gene flow of a clonal lineage of *P. striiformis* from Uzbekistan to Xinjiang, China, suggesting that pathogen movement may extend beyond national borders [81]. These findings support the development of coordinated transboundary pathogen surveillance and standardized monitoring systems across Central Asian countries.

Second, plant health policies should focus more on molecular diagnostics and routine pathogen monitoring. Recent studies in Kazakhstan demonstrate the feasibility of combining traditional molecular diagnostics with new sequencing technologies. Pozharsky et al. (2025) compared LAMP, real-time PCR, and targeted nanopore sequencing for the detection of *Erwinia amylovora* in plant materials collected from Kazakhstan and Kyrgyzstan [36]. Similarly, Maltseva et al. (2025) used systematic molecular monitoring to identify two *E. amylovora* genotypes affecting cultivated orchards and assessed the potential threat to wild *Malus sieversii* reservoirs in southern Kazakhstan [85]. For viral pathogens, Aubakirova et al. (2025) developed a nanopore workflow for monitoring grapevine viroid in Kazakhstan [86]. These studies demonstrate that investments in regional diagnostic laboratories, molecular surveillance capacity, and harmonized diagnostic protocols can significantly improve early detection and phytosanitary decision-making.

Third, the evidence suggests that disease-resistant germplasm and molecular breeding should remain a key research priority. The strong expression of wheat rust resistance, molecular markers, quantitative trait loci, and pathogen virulence indicate the importance of integrating plant pathology with breeding programs. However, recent studies showing changes in pathogen virulence suggest that selection for resistance should not rely solely on individual resistance genes. Instead, breeding programs should prioritize combinations of resistance genes, resistance of resistant adult plants, and continuous assessment of resistance in the face of changing pathogen populations. The development of molecular and genomic approaches in a regional research system provides an important basis for such strategies.

Fourth, sustainable disease management should receive greater policy attention. The evidence base is increasingly supporting the use of biological and environmentally safer alternatives to traditional chemical control. For example, studies on beneficial microorganisms in Uzbekistan have investigated the plant growth-promoting properties of endophytic bacteria isolated from xerophytic plants adapted to arid environments [87]. Such studies suggest that national plant health strategies can increasingly support locally adapted biological and low-toxicity technologies as components of integrated disease management.

Overall, the evidence from the data set suggests that Central Asian plant health policies need to shift to a regionally coordinated, prevention-oriented, and technology-enabled system. Priority areas include: (i) surveillance and early warning systems for transboundary pathogens, (ii) harmonized molecular diagnostic capabilities, (iii) monitoring pathogen populations and virulence, (iv) breeding for disease resistance, (v) development of locally adapted biological and low-toxicity control agents, and (vi) integration of artificial intelligence, remote sensing, drones, and precision agriculture into disease monitoring and management. Such an integrated strategy will allow for more effective translation of research findings into practical phytosanitary protection and sustainable crop production across the region.

### 4.4. Limitations

There are several limitations to interpreting the results of this bibliometric study. One important limitation is that the analysis was based only on publications indexed in Scopus; therefore, relevant studies published in regional journals, local language journals, conference proceedings, or databases not covered by Scopus may have been overlooked. This limitation may be particularly relevant for Central Asia, where plant disease research is conducted in multiple countries and languages. The geographic location of the recent studies in Kazakhstan and Uzbekistan, as well as the available indexed literature, suggests that the available literature may not fully reflect the plant pathology research capabilities of all five Central Asian countries. 

A parallel search of Web of Science and relevant regional or national bibliographic databases was not performed in this study. Therefore, the extent of database overlap, the number of records uniquely indexed outside Scopus, and the magnitude of potential country- or language-specific underrepresentation cannot be quantified directly. This limitation may introduce systematic differences in the observed publication distribution, particularly when comparing countries with different publication practices and levels of international database visibility. Future bibliometric studies should therefore integrate Scopus with Web of Science and relevant regional and national databases to assess database overlap and provide a more comprehensive representation of plant pathology research in Central Asia.

Another limitation concerns the fact that the dataset represents the literature available up to 6 July 2026, meaning that publication records for 2026 are incomplete. As a result, the emergence of recent topics such as molecular surveillance, genomic diagnostics, and artificial intelligence should be interpreted with caution, rather than as clear annual trends. For example, recent studies on *Erwinia amylovora* compared LAMP, real-time PCR, and nanopore sequencing for pathogen detection in Kazakhstan and Kyrgyzstan, while nanopore sequencing has also been proposed for grapevine viroid surveillance in Kazakhstan [36,74]. These examples illustrate rapidly evolving methodological directions, but their recent appearance in datasets does not mean that they have already become regional research priorities.

A further limitation is that bibliometric indicators such as publication number, citation count, co-citation citations, and keyword frequency are influenced by publication age, journal visibility, international collaboration, and indexing practices. As a result, highly cited studies should not automatically be interpreted as having great scientific or practical value. This is particularly important for plant disease research, where the scientific importance of a pathogen may depend not only on citation rates but also on its geographical distribution and economic impact. For example, molecular surveillance studies of *E. amylovora* have shown reservoirs of infection in abandoned orchards and wild *Malus sieversii* populations in South Kazakhstan and Kyrgyzstan, while studies of *Puccinia striiformis* have shown gene flow from Uzbekistan to Xinjiang, suggesting that the epidemiological importance of this pathogen may extend beyond national publication patterns [36,81]. 

Finally, the study maps the structure and evolution of the scientific literature, but bibliometric evidence alone cannot determine the effectiveness of disease management technologies in field conditions. Recent studies show that promising molecular, biological, and technological approaches still require validation in different environments and pathogen populations. Therefore, future studies should complement bibliometric mapping with systematic field assessments, standardized epidemiological data sets, and comparative assessments of disease management technologies across Central Asian agroecosystems.

### 4.5. Database Coverage and Interpretation of Country-Level Differences

The geographical distribution of publications should be interpreted in the context of the database coverage underlying this analysis. Kazakhstan and Uzbekistan accounted for the largest numbers of publications within the Scopus-indexed dataset. However, these results should not be interpreted as definitive evidence that these countries have the highest absolute research productivity in plant pathology among the five Central Asian countries. The observed distribution may reflect a combination of research activity, institutional capacity, international collaboration, publication practices, language of publication, and differences in database indexing and visibility. In particular, research published in regional journals, Russian-language or other non-English publications, and local conference proceedings that are not indexed in Scopus may be underrepresented. Such underrepresentation may also differ among countries and could therefore influence the observed geographical distribution. Consequently, the country-level results describe the distribution of internationally indexed research retrieved from Scopus rather than the complete research output of Central Asia.

## 5. Conclusions

This bibliometric study provides a comprehensive assessment of the development, structure, and new directions of plant disease and phytopathogen research in Central Asia, based on 505 Scopus-indexed publications published between 1994 and 2026. The findings indicate a steady expansion of the research field, with an annual growth rate of 11.65%, but scientific outputs are unevenly distributed across the region. Kazakhstan and Uzbekistan are the leading contributing countries within the Scopus-indexed dataset, demonstrating their central role in shaping the regional plant pathology research landscape.

Scientific mapping analyses identified three primary and interconnected research areas: (i) wheat disease resistance and molecular breeding, (ii) plant pathogens, fungal biodiversity, and disease management, and (iii) plant-associated microorganisms, microbiology, and phylogenetic characterization. Each area also reveals specific research gaps. Regarding wheat disease resistance and molecular breeding, the fluctuating virulence of pathogen populations indicates that resistance strategies must shift from reliance on individual resistance genes toward combined resistance and continuous assessment against evolving pathogen populations. In the areas of pathogen diversity, fungal biodiversity, and disease management, molecular identification and phylogenetic characterization have expanded knowledge of pathogen diversity; however, their integration into standardized diagnostics, routine surveillance, and locally adapted disease management remains insufficient. Although research on plant-associated microorganisms demonstrates the potential of beneficial microbes and microbiome-based approaches, their application to sustainable disease management and the translation of microbial diversity data into practical technologies remain in the early stages. 

Temporal analysis further identified microbiome-related research and deep learning-based approaches as emerging but still nascent topics. Given their limited publication volume and the incomplete coverage of 2026, these findings should not be interpreted as evidence of established or widespread research directions. Nevertheless, their recent appearance indicates potential for further development, particularly through the integration of high-throughput microbial profiling, molecular diagnostics, computer vision, and other data-driven approaches.

At the regional level, the findings demonstrate increasing international connectivity but also persistent disparities in research capacity and collaboration among the five Central Asian countries. The existing literature remains relatively fragmented, with monitoring efforts often focused on individual pathogens or countries and limited evidence of standardized, cross-border surveillance and data sharing. Future research should therefore prioritize stronger intra-regional and international collaboration, harmonized diagnostic and surveillance protocols, continuous monitoring of pathogen populations and virulence, durable disease-resistance breeding, and locally adapted biological and low-toxicity disease-management technologies. An integrated regional pathogen monitoring and early warning framework, supported by genomic surveillance and emerging digital technologies, could strengthen preparedness for transboundary phytopathogenic threats and contribute to sustainable crop production and food security across Central Asia.

## Figures and Tables

**Table 1:** Descriptive overview of the study.

Description	Results
Timespan	1994 to 2026
Sources	253
Documents	505
Annual growth rate	11.65%
Document average age	6.26
Average citations per document	13.94
References	87,610
Keywords	2312
Author keywords	1740
Authors	2485
Authors of single-authored docs	22
Single-authored docs	26
Co-authors per docs	6.3
International co-authorships	33.66%

**Table 2:** Most influential publication sources based on number of papers.

Journals	Articles	h-Index	TC
*E3S Web of Conferences*	23	52	22
*Online journal of biological sciences*	17	23	107
*Acta horticulturae*	14	77	177
*Bio web of conferences*	14	21	15
*Plant disease*	12	141	238
*Sabrao journal of breeding and genetics*	12	25	53
*Journal of plant pathology*	11	62	63
*Plants*	11	146	134
*IOP Conference series: Earth and Environmental Science*	9	66	28
*Caspian journal of Environmental Sciences*	7	20	22

Note: TC—total citations.

**Table 3:** Corresponding author’s countries.

Country	Articles	Articles (%)	SCP	MCP	MCP (%)	TLS
Kazakhstan	143	28.31683	114	29	20.27972	76
Uzbekistan	82	16.23762	69	13	15.85366	54
China	25	4.950495	7	18	72	39
Turkey	20	3.960396	6	14	70	53
USA	19	3.762376	11	8	42.10526	52
Kyrgyzstan	13	2.574257	9	4	30.76923	22
Germany	12	2.376238	2	10	83.33333	38
Korea	7	1.386139	2	5	71.42857	13
Poland	6	1.188119	2	4	66.66667	20
Iran	4	0.792079	2	2	50	9

Note: SCP—Single-Country Publications; MCP—Multiple-Country Publications; TLS—Total Link Strength.

**Table 4:** Top 10 most productive authors in research on plant diseases and phytopathogenic threats.

Rank	Authors	Articles	Citations	Articles Fractionalized	Country
1	Kokhmetova, Alma	22	247	3.51	Kazakhstan
2	Morgunov, A.	18	355	2.52	Kazakhstan
3	Özer, Göksel	18	124	2.98	Türkiye
4	Gritsenko, Dilyara	15	49	1.93	Kazakhstan
5	Egamberdieva, D	14	827	4.38	Uzbekistan
6	Rsaliyev, Aralbek	14	119	2.89	Kazakhstan
7	Dutbayev, Yerlan	11	43	1.62	Kazakhstan
8	Pozharskiy, A	11	37	1.41	Kazakhstan
9	Dababat, Abdelfattah A.	10	125	1.46	Türkiye
10	Doolotkeldieva, T	10	93	4.39	Kyrgyzstan

**Table 5:** Co-authorship profile.

Author	TLS	Cluster	Publications	Example
Gritsenko, D	35	2	15	[36]
Kokhmetova A	26	1	22	[37]
Pozharskiy, A	22	2	11	[38]
Nizamdinova G	22	2	9	[39]
Özer, Goksel	5	1	18	[40]
Keishilov Z	10	1	8	[41]
Rsaliyev A	15	3	14	[42]
Rsaliyev Sh	11	3	8	[43]

Note: TLS—Total Link Strength.

## Data Availability

No new data were created or analyzed in this study. Data sharing is not applicable to this article.
